# Revising the hygroscopicity of inorganic sea salt particles

**DOI:** 10.1038/ncomms15883

**Published:** 2017-07-03

**Authors:** P. Zieger, O. Väisänen, J. C. Corbin, D. G. Partridge, S. Bastelberger, M. Mousavi-Fard, B. Rosati, M. Gysel, U. K. Krieger, C. Leck, A. Nenes, I. Riipinen, A. Virtanen, M. E. Salter

**Affiliations:** 1 Department of Environmental Science and Analytical Chemistry, Stockholm University, SE-10691 Stockholm, Sweden; 2 Bolin Centre for Climate Research, SE-10691 Stockholm, Sweden; 3 Department of Applied Physics, University of Eastern Finland, FI-70211 Kuopio, Finland; 4 Laboratory for Atmospheric Chemistry, Paul Scherrer Institute, CH-5232 Villigen, Switzerland; 5 Institute for Atmospheric and Climate Science, ETH Zürich, CH-8092 Zürich, Switzerland; 6 Department of Meteorology, Stockholm University, SE-10691 Stockholm, Sweden; 7 School of Earth and Atmospheric Sciences and Chemical and Biomolecular Engineering, Georgia Institute of Technology, Atlanta, Georgia 30332-0100, USA; 8 Institute of Chemical Engineering Sciences, Foundation for Research and Technology Hellas, GR-26504 Patras, Greece; 9 Institute or Environmental Research and Sustainable Development, National Observatory of Athens, GR-15236 Palea Penteli, Greece

## Abstract

Sea spray is one of the largest natural aerosol sources and plays an important role in the Earth’s radiative budget. These particles are inherently hygroscopic, that is, they take-up moisture from the air, which affects the extent to which they interact with solar radiation. We demonstrate that the hygroscopic growth of inorganic sea salt is 8–15% lower than pure sodium chloride, most likely due to the presence of hydrates. We observe an increase in hygroscopic growth with decreasing particle size (for particle diameters <150 nm) that is independent of the particle generation method. We vary the hygroscopic growth of the inorganic sea salt within a general circulation model and show that a reduced hygroscopicity leads to a reduction in aerosol-radiation interactions, manifested by a latitudinal-dependent reduction of the aerosol optical depth by up to 15%, while cloud-related parameters are unaffected. We propose that a value of *κ*_s_=1.1 (at RH=90%) is used to represent the hygroscopicity of inorganic sea salt particles in numerical models.

## Abstract (web summary)

Sea spray, one of the largest natural aerosol sources, plays an important role in the Earth’s radiative budget. Here the authors show that the ability of sea salt particles to take up water is smaller than for pure salt, with implications for the parameterization of the direct aerosol radiative effect.

Sea spray aerosol is produced over the open oceans that cover more than 70% of the Earth’s surface and it is therefore one of the largest natural aerosol sources^1^. These particles consist of a mixture of inorganic salts, here termed sea salt particles, and organic compounds and they are generated at the ocean surface (we term the mixture of inorganic and organic compounds sea spray aerosol). Here, wind-driven breaking waves are regarded as the main driver of sea spray aerosol production^2^. Breaking waves entrain air into the water, which rises as bubbles to the surface. Droplets are injected into the atmosphere when these bubbles burst and two major droplet types can be identified. A large number of submicrometre size droplets are formed by the bursting of the thin bubble cap, the so called film droplets^3^,^4^. The bursting of the bubble cap is followed by the ejection of fewer, so called jet droplets, that are produced by the collapse of the remaining air bubble cavity. Jet droplets are mainly found in the supermicrometre size range^5^.

The ability of particles to take up water, termed hygroscopicity, is determined by the particle’s size and chemical composition. It is an important quantity as it influences the magnitude of the direct aerosol radiative forcing^6^ since ambient aerosol optical properties will depend on the ambient relative humidity (RH)^7^. The hygroscopicity also affects the particle’s efficiency to act as cloud condensation nuclei (CCN) (ref. 8). Particle size and mass changes due to water uptake are also important for the kinematics of particle deposition fluxes, that is, influencing the atmospheric residence times of sea salt particles in the marine boundary layer^5^. The chemistry of the marine boundary layer is also influenced by the particle’s hygroscopicity by increasing the water volume available for absorption of gases and subsequent heterogeneous chemical reactions^9^.

It is commonly assumed that the hygroscopicity of sea salt particles is very similar to that of pure sodium chloride (NaCl; see page 54 of Lewis and Schwartz^5^, and references therein). There are a large number of expressions relating RH to the growth of sea salt particles and, according to Lewis and Schwartz^5^, most of these expressions agree to within a few per cent with the measurements of Tang *et al*.^10^ for NaCl. In their work, Tang *et al*.^10^ compared the hygroscopicity of particles generated from ambient seawater to those generated from NaCl using an electrodynamic balance (EDB). The conclusion of this study was that no significant difference in hygroscopicity exists between particles generated from seawater and NaCl aqueous solutions.

The reduction in hygroscopic growth relative to NaCl that is often observed in ambient measurements in the remote marine atmosphere is generally attributed solely to the contribution of internally mixed organic substances^11^. Unfortunately, this assumption is not easy to validate for a number of reasons. First, ambient measurements of nascent sea spray aerosol are always influenced by aerosols from other sources. Second, nascent sea spray aerosol also contains organic matter. In addition, laboratory measurements of the hygroscopicity of sea salt often suffer from significant diversity in the measured values, lack of detail in measurement descriptions, and differences in particle production techniques (see Supplementary Table 1 for an overview).

The variability, in terms of water uptake, of aerosol particles among major large-scale models is still large^12^. One reason for this disagreement is differences in the assumptions made by each hygroscopicity parameterization implemented. This highlights the need to continuously constrain hygroscopicity parameterizations.

Within this work, we have performed a large suite of well-controlled laboratory experiments on the water uptake of sea salt particles to reduce the uncertainty in their hygroscopicity. The hygroscopicity measurements covered the sub- and super-micrometre particle size range using a hygroscopic tandem differential mobility analyser (HTDMA) and an EDB. In addition, measurements of the water activity of the bulk solutions were also conducted. Sea salt particles were generated using both a sea spray aerosol chamber and a nebulizer, using aqueous solutions of pure NaCl and artificial seawater (to avoid the effect of organics present in natural seawater). Using these three techniques, we demonstrate that the hygroscopic growth of inorganic sea salt is, in terms of diameter change, 8–15% lower than NaCl, most likely due to the difference in chemical composition, especially the presence of hydrates. Since measurements made using a HTDMA are sensitive to particle shape, the dynamic shape factor of the generated particles was also measured directly. These measurements demonstrated that the shape of the particles was dependent on both their chemical composition and the method with which they were generated. To yield insights into the mechanisms controlling the hygroscopicity at different particles sizes, we compared the measured hygroscopic growth factors to predictions made by thermodynamical models using the measured inorganic chemical composition as their input. Finally, we have tested the significance of our results by reducing the hygroscopic growth of the sea salt component in a general circulation model. Reducing the hygroscopic growth resulted in a reduction in aerosol-radiation interactions, while cloud-related parameters were not affected.

## Results

### The shape of sea salt and NaCl particles

**Figure 1:**
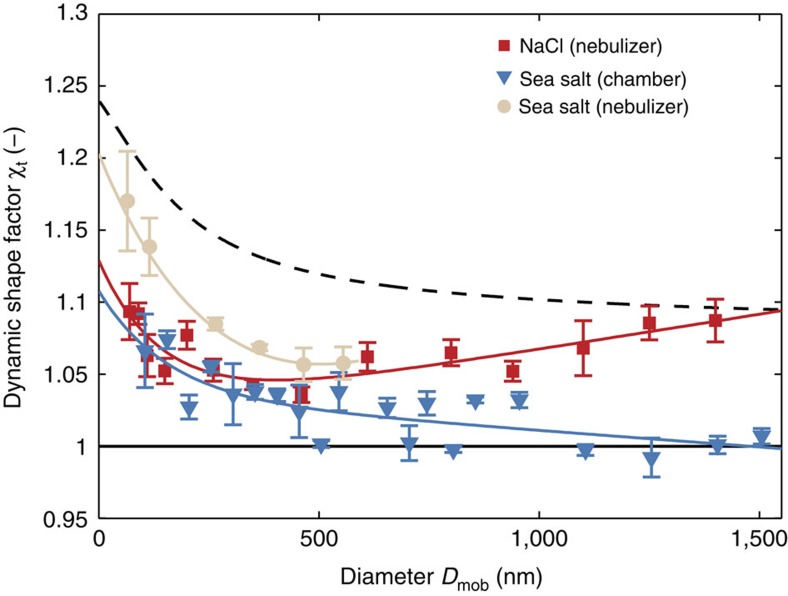
Shape factor measurements of inorganic sea salt and NaCl particles. Dynamic shape factor *χ*_t_ versus mobility diameter of sodium chloride (NaCl) and inorganic sea salt generated both by a nebulizer and the sea spray chamber. Error bars denote the s.d. of repeated measurements. The corresponding lines represent exponential decaying fits. The black dashed and solid curves show theoretical values of *χ*_t_ for perfect cubes and perfect spheres, respectively.

The measured values of the dynamic shape factor, *χ*_t_, for sea salt particles generated by the sea spray simulation chamber and by the nebulizer, as well as the NaCl particles generated by the nebulizer, are depicted in Fig. 1. The curves corresponding to perfect spheres (unity) and perfect cubes (using the Dahneke adjusted-sphere interpolation^13^ as detailed in Biskos *et al*.^14^) are also shown. The lines in Fig. 1 represent exponential decaying fits applied to the individual measurement (see Supplementary Table 2 for equation and fit-coefficients). The sea salt particles generated by the nebulizer are closer to perfect cubes than the sea salt particles generated by the sea spray chamber, indicating that the generation method influences particle shape. Interestingly, the sphericity of the sea salt particles generated in the sea spray chamber increased with particle size. The NaCl particles generated by the nebulizer, on the other hand, transitioned from almost cubic particles at supermicron particle sizes to more spherical shapes at smaller particle sizes, resembling the chamber-generated sea salt for particles smaller than 150 nm. This observation is in accordance with the electron micrographs of Zelenyuk *et al*.^15^, who observed rounder edges on smaller nebulized-NaCl particles, which corresponds to smaller shape factors. The shape effects on particle size have been corrected for in all HTDMA results presented from here onwards.

### The hygroscopicity of inorganic sea salt particles

**Figure 2:**
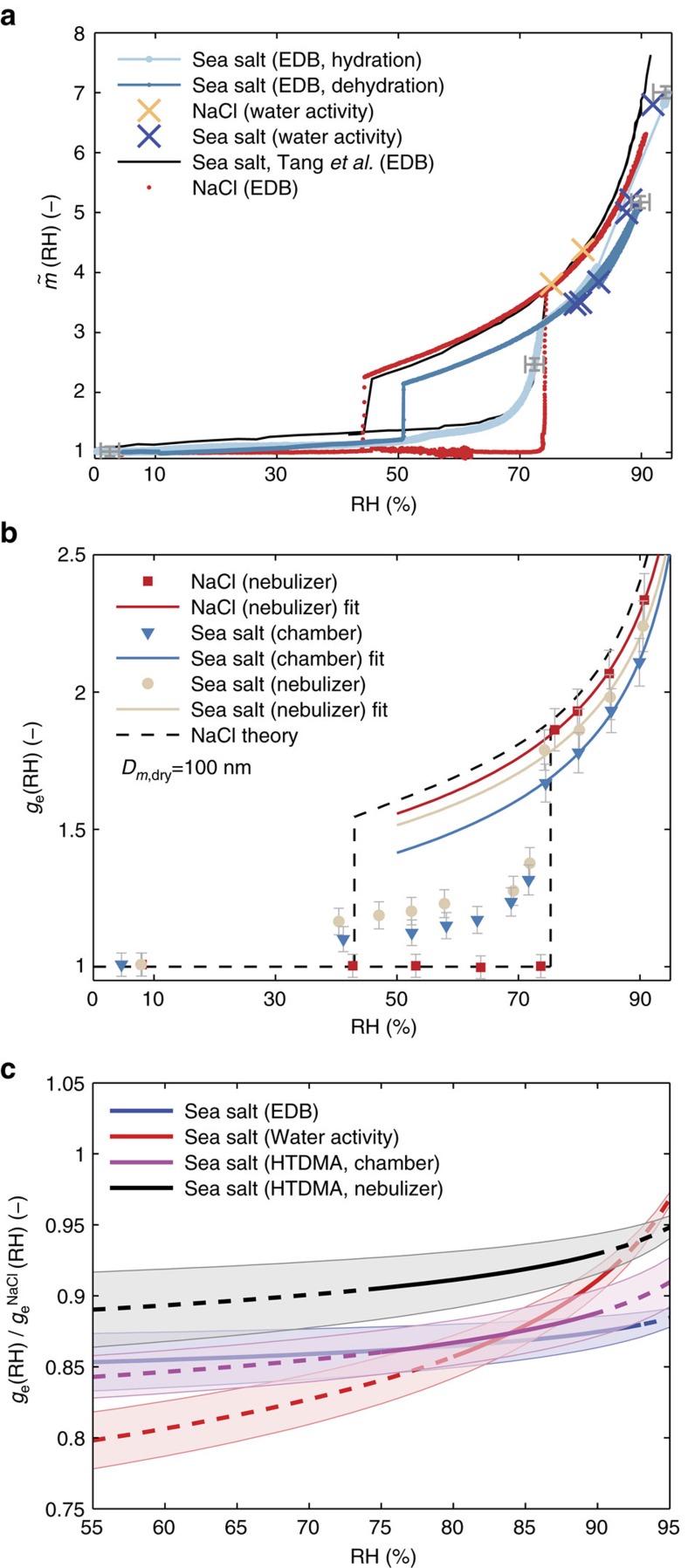
Hygroscopicity measurements of inorganic sea salt and NaCl particles. (**a**) Mass growth factor 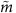
(RH) versus RH for artificial sea salt and NaCl determined from EDB and water activity measurements. Results from Tang *et al*.^10^ for ambient seawater measurements are shown as reference. The uncertainties (s.e.m.) for the EDB are shown at 4 selected RH-values. The hydration and dehydration branches are shown specifically only for the EDB measurement of the artificial sea salt. (**b**) Hygroscopic growth factor *g*_e_(RH) versus RH at *D*_m,dry_=100 nm measured by the HTDMA for NaCl, for inorganic sea salt originating both from the nebulizer and the sea spray chamber. Error bars denote the propagated measurement uncertainty. The measurements of the upper hysteresis branch were fitted by a two parameter *γ*-fit. The dashed black line represents theoretical calculations for NaCl (refs 1, 22). (**c**) Ratio of *g*_e_(RH) (calculated to bulk values) related to the measurement of ambient sea salt by Tang *et al*.^10^ which is identical to the predicted and measured (water activity) values for pure NaCl. Shaded areas for the EDB measurements give uncertainty due to the assumed RH-dependent density, while the shaded areas for the HTDMA state the variation of the measurements at the three distinct dry diameters after the values were back-calculated to bulk values. Dashed lines indicate RH-ranges where the data was extrapolated. Note the different x-scale in **c**.

The EDB and water activity measurements target the bulk properties of the inorganic salt. Figure 2a presents the mass growth factor (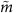, defined as the ratio between wet and dry particle masses) as determined by these techniques for the artificial sea salt and NaCl, along with the findings of Tang *et al*.^10^ for ambient sea salt particles. The EDB results show that the sea salt particles take up water at intermediate RH-values below 70% in accordance with the observations of Tang *et al*.^10^. The sea salt also exhibits some water uptake at RH∼10–15% and RH∼55%, caused by salt components other than NaCl (for example, MgCl_2_ and CaCl_2_). These partially dissolved states, known as eutectics or mutual deliquescence^16^, were also observed by Tang *et al*.^10^ and recently by Gupta *et al*.^17^. NaCl exhibits its main deliquescence RH (DRH) at RH=74.3±1.5% (complete dissolution of NaCl), while the main DRH of the inorganic sea salt mixture is slightly shifted to lower RH (∼73.5%). At RH’s above 80%, both the EDB and the water activity measurements of the inorganic sea salt mixture exhibit approximately 15–20% smaller mass growth factors relative to NaCl and the observations of sea salt made by Tang *et al*.^10^. At RH>90% the water activity measurements of the artificial sea salt show slightly larger values of 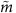 than the EDB, probably due to the increased uncertainty of the RH-sensor. However, the values are still clearly below the measurements of sea salt determined by Tang *et al*.^10^ and our measurements of the mass growth of NaCl.

The hygroscopic growth of the sea salt and NaCl particles in the submicrometre size range was determined using the HTDMA data and includes the measured dynamic shape factors (Fig. 1). Figure 2b depicts the hygroscopic growth factor *g*_e_(RH) (see equation (2)) for 100 nm particles. The DRH of pure 100 nm NaCl particles was between 73.7% (last measurement in crystalline state) and 76.0% (first measurement in completely deliquesced state), which is in agreement with both our EDB measurements of NaCl as well as those of Tang *et al*.^18^. HTDMA (chamber and nebulizer experiments) and EDB show the same main DRH for the sea salt particles, suggesting no effect of the particle generation method on the DRH which is also in accordance with previous findings^19^. At RH’s above 40%, the particles generated by the nebulizer consistently show an increased hygroscopic growth compared to the particles generated in the sea spray chamber (see also Table 1). However, regardless of the generation method the sea salt particles always exhibit hygroscopic growth at relative humidities below the DRH of NaCl.

The values of *g*_e_(90%) reported in the literature for NaCl particles with *D*_m,dry_=100 nm range from 2.29 (ref. 20) to 2.46 (ref. 21; see Supplementary Table 1). Our measured values for NaCl agree well with Gysel *et al*.^20^ who used almost the same dynamic shape factor correction (*χ*_t_=1.08) that we have determined directly (Fig. 1). This highlights how important the assumption of a shape factor is to the overall uncertainty in *g*_e_ measurements using HTDMAs, and may at least partly explain the large variation among the different studies. The values of *g*_e_(90%) for NaCl measured here are generally consistent, although they are 3.5% lower than the theoretical predictions by Topping *et al*.^22^.

To directly compare all three hygroscopicity measurements, the bulk mass hygroscopic growth factors (Fig. 2a) were converted to diameter growth factors (*g*_e_(RH)) by assuming two different RH-dependent densities (see Methods section). The HTDMA measurements for the 100 nm particles were then converted to bulk *g*_e_(RH) values by approximately accounting for the Kelvin effect using the parameterization of Lewis^23^. Finally, all the measurements were fitted by the same two parameter *γ*-fit as used for the HTDMA humidograms (Fig. 2b). Figure 2c shows the difference of the bulk *g*_e_(RH) obtained in this manner for the sea salt to the *g*_e_(RH) of NaCl (see also Supplementary Fig. 1 for the absolute humidogram of the bulk *g*_e_(RH)). It is evident that the hygroscopic growth factors of the inorganic sea salt measured here are up to 15% lower than the values measured by Tang *et al*.^10^ and those for NaCl obtained using an EDB. The bulk values of *g*_e_(RH) derived independently from HTDMA and EDB agree surprisingly well and show almost the same discrepancy compared to the values of Tang *et al*.^10^ (see Fig. 2c and also Supplementary Fig. 1). The bulk water activity measurements of the inorganic sea salt are in the same range as the HTDMA and EDB measurements but exhibit a slightly smaller discrepancy compared to Tang *et al*.^10^ at elevated RH (8–10%).

**Figure 3:**
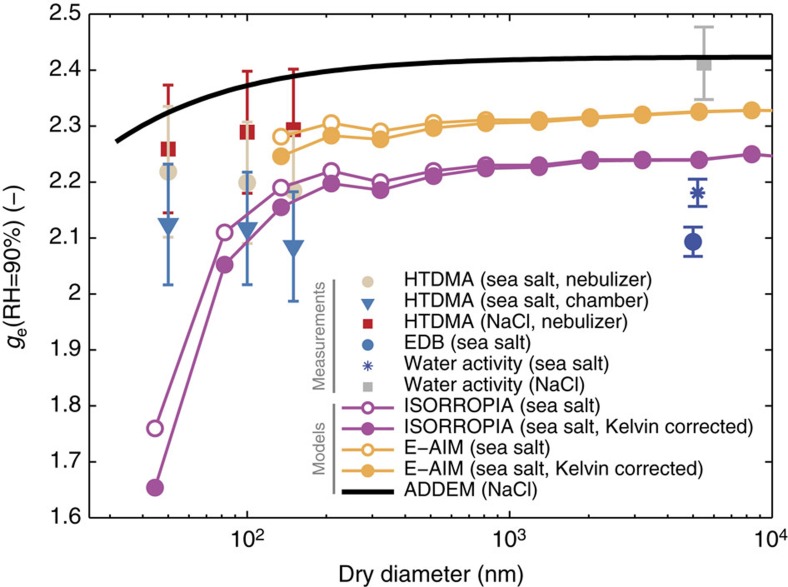
Hygroscopicity measurements compared to thermodynamical model predictions. Modelled and measured hygroscopic growth factor *g*_e_(RH) versus dry particle diameter at RH=90%. The values for E-AIM and ISORROPIA-II used the size-dependent chemical compositions measured by Salter *et al*.^24^. The Kelvin effect was accounted for using the modified correction by Lewis^23^. Error bars of HTDMA measurements denote propagated measurement uncertainties, while error bars of EDB and water activity measurements denote the range for the assumptions on the RH-dependent density when converting to *g*_e_(RH).

The hygroscopicity of the inorganic sea salt particles smaller than 150 nm increases with decreasing particle size (see Fig. 3, where the *g*_e_(RH) is shown at RH=90% for all three dry diameters measured with the HTDMA). The trend in the values of *g*_e_(RH) as a function of particle size is very similar for the nebulizer and the sea spray chamber experiment, exhibiting a similar linear behaviour (with respect to *D*_m,dry_) with an off-set difference of Δ*g*_e_(RH)=0.08. Figure 3 also presents the comparison to the modelled hygroscopic growth factors for NaCl and the inorganic sea salt at RH=90% as a function of dry particle diameter, accounting for the size-dependent composition^24^ and approximating the Kelvin effect using the parameterization by Lewis^23^, where relevant. Further details on the different models used can be found in the method section. The ADDEM model^22^ prediction for NaCl is consistently higher than all the observations, but agrees with the water activity measurements of NaCl. Further, it exhibits a dependence on particle size that is totally opposite to that which we observe. Predictions with the online version of E-AIM also yielded significantly larger values of *g*_e_(RH=90%) than those measured for the inorganic sea salt. E-AIM predicts a slight decrease in *g*_e_(RH=90%) with decreasing particle size due to the increased contribution of calcium, but the *g*_e_(RH=90%) at the lower sizes could not be predicted with the online version of E-AIM due to an ion imbalance caused by predicted precipitation of CaCO_3_ and MgCO_3_.

The ISORROPIA-II model predicted lower values of *g*_e_(RH=90%), that are closer to the measured values at larger particle sizes, than both ADDEM and E-AIM. Despite this improvement, the model still poorly resolves the measured hygroscopicity of the smallest particles measured. In difference to both ADDEM and E-AIM, the ISORROPIA-II calculations allowed CaSO_4_ to precipitate out of solution. For the smallest particles considered here, it may be possible that the size of the precipitate core that would result may be small enough to promote complete solubility of CaSO_4_, even if supersaturated^25^. If true, this would suggest that the reduction in hygroscopicity of ISORROPIA-II relative to the measured values, which is mostly from a reduction of soluble material as CaSO_4_ precipitates out, is a result of curvature effects in precipitate solubility that are not included in the model calculations.

Since none of the models capture the observed increase in hygroscopicity with decreasing particle size in the submicrometre size range and given that this size-dependence is different from what would be expected based on the simple Kelvin effect for water vapour equilibration, our results indicate that additional size-dependent phenomena are taking place. Such phenomena could include the enhancement of the solubility of the sea salt components, as discussed in the case of ISORROPIA-II, or a decrease of the surface tension with decreasing particle size (see for example, Cheng *et al*.^26^, Werner *et al*.^27^ and Ruehl *et al*.^28^). If the surface composition is driving the water uptake at these small sizes, only minute amounts of surface-tension-reducing substances are needed to result in a notable effect^29^. Salter *et al*.^24^ observed Ca^2+^-enrichment in nascent sea spray aerosols using both the same artificial sea salt solution used in this study and one ambient seawater sample. Both seawater samples showed the same size-dependence and magnitude in Ca^2+^-enrichment, independent of the amount of organic carbon in the seawater. As such Salter *et al*.^24^ concluded that the enrichment in Ca^2+^ may not require the presence of organics. Taken further, these observations may, at least partially, explain our observations of increasing hygroscopic growth with decreasing particle size in submicrometre inorganic sea salt particles.

Table 1 summarizes the measured values of *g*_e_ (RH=90%) and lists the corresponding *κ*_s_ values. The values for *κ*_s_ were calculated according to Petters and Kreidenweis^30^ at RH=90% assuming the surface tension of water. A decrease in *κ*_s_ with increasing particle size is evident for submicrometre particles. As mentioned above, the value of *κ*_s_ for the supermicrometre range (EDB) can only be given as a range due to the uncertainty of the dry particle density. However, the supermicrometre *κ*_s_ values are consistent with the submicrometre values, which adds support to the view that the hygroscopicity of inorganic sea salt particles is not equal to that of NaCl and instead is measurably reduced. For simplified applications, such as model parameterizations, we suggest that a value of *κ*_s_=1.1 at RH=90% (the mean value of the EDB and HTDMA chamber measurements) is used to represent inorganic sea salt aerosol particles.

### Impact of a reduced hygroscopicity within a climate model

To test the impact of the reduced inorganic sea salt hygroscopicity within a general circulation model, three ECHAM6-HAM2 model runs were conducted using three different hygroscopicity parameter *κ*_s_ values for the inorganic sea salt component. The values chosen (*κ*_s_=1.5, 1.3 and 1.1) cover the observed *κ*_s_ values from our study, with 1.5 representative of NaCl and 1.3 and 1.1 representative of the HTDMA measurements of particles generated using the nebulizer and sea spray chamber, respectively. It should be noted that in the default configuration of ECHAM6-HAM2 the hygroscopicity of the inorganic sea salt aerosol component is assumed to be the same as that of NaCl. However, the exact value used, *κ*_s_=1.12, is not that of NaCl (the correct value for NaCl should be *κ*=1.5 at RH=90%). The incorrect value for NaCl that is currently implemented in the model is taken from Petters and Kreidenweis^30^ who erroneously transcripted a value from Koehler *et al*.^31^. Interestingly, Zhang *et al*.^32^ noted an underprediction of the water uptake by pure NaCl compared to AIM^33^ in the ECHAM6-HAM2 model but did not elaborate further on it. As such, the water uptake by sea salt aerosol in ECHAM6-HAM2 seems too low based upon the range of *κ*-values for NaCl published in the literature and by sheer coincidence matches closely our measured value of *κ*_s_.

**Figure 4:**
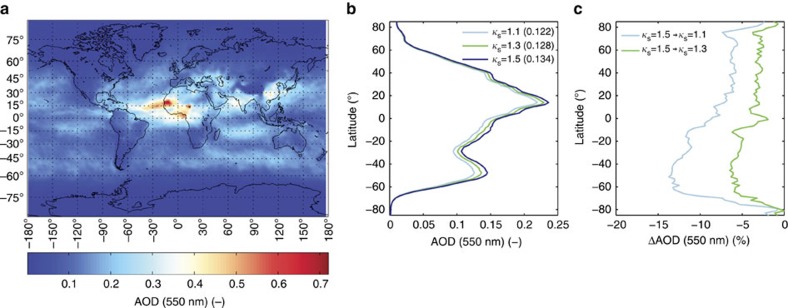
Impact of reduced inorganic sea salt hygroscopicity within a general circulation model. (**a**) Global map of the aerosol optical depth (AOD) at *λ*=550 nm with the hygroscopicity of the inorganic sea salt component set to *κ*_s_=1.5 (NaCl). (**b**) Latitudinal mean of the AOD(550 nm) for *κ*_s_=1.5, 1.3 and 1.1. Global mean values of AOD for each model run are given in parenthesis in the legend. (**c**) Percental change in AOD when decreasing the hygroscopicity of the inorganic sea salt component from 1.5 to 1.3 and 1.1, respectively. Results are shown using the sea spray source function of Gong *et al*.^34^.

A change in the hygroscopicity of sea salt aerosol will directly affect its optical properties with the largest effects on how much light it scatters^7^. Therefore, the effect of reduced hygroscopicity is expected to be manifested in a change of aerosol optical depth (AOD). Figure 4a shows a global map of the AOD at *λ*=550 nm for the model simulation where *κ*_s_ is first set to 1.5 (NaCl). Large values of AOD are shown close to arid regions (mineral dust) and densely populated areas (for example, the anthropogenic emissions from Asia). The contribution of sea salt particles is clearly visible over ocean areas, especially over the Southern Ocean. Figure 4b shows a latitudinal mean value of the AOD at the wavelength of 550 nm for the three model runs. As can be seen in Fig. 4c, changes of up to 15% in AOD were calculated over oceanic areas if the hygroscopicity parameter is reduced from *κ*_s_=1.5 (NaCl) to *κ*_s_=1.1. Other parameters that include light scattering processes by sea salt particles, such as the clear sky aerosol radiative forcing, are affected in the same way as the AOD (see Supplementary Fig. 2). We tested two independent sea spray source functions^34^,^35^ and although the latitudinal average of the AOD was different depending on the source function used, the relative changes to AOD remained almost unchanged (see Supplementary Fig. 3). A constrained hygroscopicity parameter for inorganic sea salt, as presented here, will therefore remove a systematic bias.

Indirect processes, such as the cloud droplet number concentration, remain unaffected by the change in *κ*_s_. This low sensitivity is because the marine boundary layer represents a number limited regime with respect to droplet activation. That is, the potential number of particles that can activate to cloud droplets is limited by the concentration of aerosol particles within a size regime that are large enough to activate. A reduction in *κ*_s_ from 1.5 to 1.1 is simply not sufficient to elicit a clear change in cloud droplet number (Supplementary Fig. 4).

## Discussion

We demonstrate that the hygroscopic growth factor of inorganic sea salt particles is significantly lower (8–15%) than NaCl using three independent measurement techniques. This is interesting given that NaCl is generally assumed to well represent the hygroscopicity of inorganic sea salt aerosol^5^. In fact, a range of values of hygroscopic growth of inorganic sea salt can be found in the literature (see Supplementary Table 1) from ∼2.1 (ref. 36) at *D*_dry_=100 nm and RH=90%, which is similar to the value determined in our chamber experiments, to ∼2.46 (ref. 37) at *D*_dry_=100 nm and RH=90%, which is close to (or even slightly larger) than the value for NaCl. However, these studies encompass a range of different aerosol generation techniques, different artificial sea salt solutions, assumed or unreported shape factor correction and differing dry RH. Some of the studies also lack detail in instrument calibration. These issues highlight the importance of thorough reporting of methods and collocated shape measurements with similar flow and drying conditions^38^. Given that sea salt aerosol particles take up significant amounts of water at RH<75%, due to the presence of the highly hygroscopic salts of Ca^2+^ and Mg^2+^, ensuring that the dry reference aerosol has as low an RH as possible is also essential for precise determination and possible comparison of hygroscopic growth factors.

With regards to the importance of the particle generation mechanism, we observe a clear difference in the hygroscopic growth and the dynamic shape factor of the particles generated with the plunging jet compared to the nebulizer. The reasons for this are unclear, but entraining air by impinging water from above, in a manner similar to the plunging jet deployed in our study, is likely to be more representative of breaking waves than standard nebulizers. For this reason, the hygroscopic growth of the particles generated by the sea spray chamber are likely to better reflect the hygroscopic growth of nascent sea salt aerosol and we propose that these values should be used as a baseline reference in future studies.

That the suppression of the hygroscopic growth of the sea salt aerosol below NaCl was comparable across the three independent techniques we used increases our confidence in the results. It also suggests that it is the inorganic composition of the particles that is primarily responsible for this suppression rather than organic contamination, which is often referred to as the main mechanism by which the hygroscopic growth of (ambient) sea spray is reduced in the literature^11^. In the ambient atmosphere, the contribution of organic substances may further decrease the hygroscopicity of the ambient sea spray particles^39^. In addition, we see an increasing hygroscopicity with decreasing particle size with both particle generation methods. This suggests the importance of size-dependent surface phenomena, such as curvature-enhanced solubility or surface tension reductions, in determining the hygroscopic growth of sea salt aerosol particles smaller than 150 nm. To our knowledge, this is the first report of such size-dependent hygroscopicity for particles dominated by purely inorganic sea salt components.

Inorganic sea salt components contain water even at very low RH as an integral part of the crystalline structure. Examples of hydrates which may be important are MgCl_2_·6H_2_O and CaCl_2_·10H_2_O. These sea salt hydrates are present in all measurements of sea spray aerosol including those made by Tang *et al*.^10^ who stated in their paper that 5–10 wt% water was always present in their particles at low RH. That hydrates are present in both the sea salt particles in the present study and in the measurements conducted by Tang *et al*.^10^ means that they cannot explain the difference in the hygroscopic growth between the two studies. However, if Tang *et al*.^10^ removed the contribution of water to the dry mass in their EDB measurements this may explain these differences. This observation is critical—if this residual water was removed by Tang *et al*.^10^ then their values for the hygroscopic growth of sea salt (which would not include the atmospherically relevant hydrates) have been erroneously transcripted into atmospheric models. Notably, the presence of hydrates in sea salt under atmospherically relevant conditions also has relevance beyond hygroscopicity—it is common practice to estimate the organic fraction of sea spray aerosol based on the volatility of aerosols (see for example, Modini *et al*.^21^). Since a significant fraction of what these authors measure is the decomposition of hydrates this suggests this approach should be used with caution (see recent work by Rasmussen *et al*.^40^ on sea spray volatility).

To test the implications of our observations, we used the general circulation model ECHAM6-HAM2 where only the hygroscopicity parameter of the inorganic sea salt component was changed across the range of our observations. The reduction in sea salt hygroscopicity manifested in a decrease of up to ∼15% in AOD if *κ*_s_ was reduced from 1.5 to 1.1, while indirect or cloud related effects were practically insensitive to this change. Although modelling studies have suggested that the presence of organics in sea spray acts to reduce its hygroscopicity^39^ recent measurements suggest that organic substances do not significantly reduce the hygroscopicity of nascent sea spray aerosol^41^,^42^. As such, to understand the hygroscopicity of sea spray aerosol it is critical that we first understand the inorganic fraction which dominates the water uptake. Based on our measurements we would recommend a bulk solution *κ*_s_ value of between 1.06–1.29 and a *κ*_s_ value of between 1.00–1.16 for the submicrometre range (both at RH=90%). The increased range in the suggested values for the submicrometre particles results from the apparent change in inorganic composition and the size-dependence of the water uptake. As a further simplification, ignoring the size and particle generation dependencies, we suggest that a *κ*_s_ value of 1.1 (at RH=90%) is representative of the hygroscopicity of inorganic sea salt aerosol particles.

To summarize, the hygroscopicity of inorganic sea salt is significantly lower compared to NaCl. Therefore, hygroscopicity measurements of artificial sea salt should always be the baseline reference instead of pure NaCl. Improving our understanding of the behaviour of the inorganic fraction of sea spray aerosol is a prerequisite for understanding the hygroscopicity of the complex mixture that is sea spray aerosol, including the effect of organic substances. Within this context, inorganic hydrates, that are present even at low relative humidities, are clearly important for sea spray hygroscopicity. Finally, our results have relevance beyond hygroscopicity—it is common practice to estimate the organic fraction of sea spray aerosol based on their volatility. Since most of what these authors measure may be the decomposition of hydrates this suggests this approach may be invalid.

## Methods

### Sea salt particle generation methods

Two methods were used to generate sea salt aerosol in the laboratory. First, a sea spray chamber utilizing a plunging jet to entrain air from above the water surface into the seawater was used^43^,^44^. The bursting of the rising air bubbles generated sea salt aerosol particles that were subsequently sampled. The cleaning protocol of the chamber was identical to that described in Salter *et al*.^24^. A schematic of the set-up can be found in the Supplementary Material (Supplementary Fig. 5). Second, conventional medical nebulizers (Medix, Clement Clarke) were used. These are standard unvented jet nebulizers^45^, where pressurized particle free air is entrained from below into the small sterile sample reservoir.

An artificial sea salt mixture (Sigma Aldrich, S9883; mass fraction: 55% chloride (Cl^−^), 31% Na^+^, 8% sulfate (SO_4_^2−^), 4% Mg^2+^, 1% K^+^, 1% Ca^2+^ and <1% other) was used for both the chamber and nebulizer experiments. It is representative of the inorganic mass fraction of most oceanic seawater since the major elements in seawater can be regarded as having almost constant proportions globally^46^. In Salter *et al*.^24^ we used both X-ray photoelectron spectroscopy and vibrational sum frequency spectroscopy to test whether surface active organic matter was present in artificial sea salt. Both surface-sensitive methods did not observe any surface active organics. In addition, the manufacturer states that no anti-caking organics are used in the production of the salt mixture. Therefore, organic substances would only have been present in similarly low concentrations (below detection limit) across all of our measurements which means any effect on the hygroscopic growth will have been negligible. Even at very low RH, sea salt contains water in the form of hydrates^47^. We have confirmed this using Fourier transform infrared spectroscopy on the bulk inorganic sea salt mixture. The density of the artificial sea salt was experimentally determined using a helium-pycnometre (AccuPyc, Micromeritics Instrument Cooperation) and found to be (2.017±0.006) gcm^−3^ (mean±s.d.). The sea salt was rehydrated to an absolute salinity of 35 g kg^−1^ in the sea spray simulation chamber using low-organic-carbon standard deionized water (MilliQ, >18.2 MΩ cm). The particles generated from this inorganic sea salt mixture are referred to as sea salt particles. In addition, control experiments were performed with pure NaCl (VWR International, 27800.360) for the nebulizer experiments.

The particles generated by the sea spray chamber and the nebulizer were sampled from a custom-made manifold that allowed a mixing of the sample flow with particle free dry air (Kaeser Kompressoren, Dental 1T, KCT110) which was followed by a silica diffusion dryer to reach low RH conditions (RH<10%). The dilution and drying rates were kept similar for all experiments to avoid drying related variability of particle shape factors that can occur during the crystallization of salt particles^38^.

### Shape factor measurements

To accurately determine the hygroscopic growth of aerosol particles using a HTDMA (see next section) knowledge of the dynamic shape factor, *χ*, is required and this was determined as follows: particles were size-selected according to their electrical mobility by a differential mobility analyzer (DMA) preceded by a Ni-63 bipolar charger. The DMA was constructed at the Paul Scherrer Institute and is of the same design as the TSI Inc. model 3801 Long DMA, which means that it was limited to selecting submicrometre particles. No impactor was used upstream of the DMA and a sheath to sample flow ratio of 10 was used.

Monodisperse classified aerosol from the DMA was passed through an aerosol particle mass analyzer (APM; Kanomax APM-II model 3601 (ref. 48)), followed by a condensation particle counter (TSI Inc., Model 3022A). The APM was programmed to scan a range of particle mass-to-charge ratios. The range was adjusted to the selected nominal mobility diameter so that singly charged or doubly charged particles of interest were selected. Singly charged particles were used for measurements for diameters below 825 nm (the single-charge upper size limit of the DMA) while doubly charged particles were selected to obtain measurements up to 1,500 nm. Doubly charged particles were also measured for particles smaller than 825 nm to validate the approach. The mode of the resulting APM mass scans was determined by fitting either asymmetric normal or lognormal functions, which were chosen according to their ability to describe the data. The fitted mode provided the single-particle mass; the s.d. of repeated measurements provided the corresponding uncertainty. In some cases of poor signal to noise, the lower one or two quartiles of the measured data were excluded from the fit. When large particle masses were selected, fluctuations in the APM mass set-point became significant during the ∼10 s residence time in the APM. Therefore, the measured APM-set-point data were smoothed with a locally weighted scatterplot smoothing (LOESS) function.

The APM selects particles of a given charge solely by mass (that is, particle shape does not influence the measurements), which allows the particle volume-equivalent diameter, *D*_e_, to be calculated as *D*_e_^3^=6*m*/(*ρπ*) if the void-free material density (*ρ*) is known. For NaCl the material density was assumed to be *ρ*=*ρ*_NaCl_=2.16 g cm^−3^. For the sea salt particle we used the experimentally determined value of *ρ*_s_=2.017 g cm^−3^ (see section above). It should be noted that *ρ*_s_=2.0 g cm^−3^ is at the lower limit of density values to retrieve reasonable shape factors with *χ*_t_ generally ≥1. The combination of a DMA and an APM makes it possible to determine the shape factor of the particles, that is, the transition regime value for the selected size, as follows^49^:


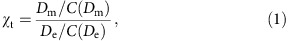


where *D*_m_ denotes the mobility diameter and *C* the Cunningham slip correction^50^.

### Hygroscopicity method I

A custom-built hygroscopicity tandem differential mobility analyser (HTDMA; University of Eastern Finland) was used to determine the hygroscopicity of submicron particles. The particles of the dry aerosol sample stream (RH<10%) were charged by a Ni-63 bipolar charger. The particles then entered the first DMA (custom-made Vienna type DMA, length 28 cm, outer radius 33 mm and inner radius 25 mm) which was set to select a nearly monodisperse particle population at three different dry mobility diameters (*D*_dry_=50, 100, 150 nm) from the initial polydisperse aerosol sample. A humidifier (Gore-tex tube, length 150 mm, custom-made) then humidified the aerosol to a controlled RH up to 95%. The aerosol stream subsequently entered a second closed-loop DMA (same type as first DMA) followed by a condensation particle counter (TSI Inc., Model 3010, sample flow 1 lpm) where the equilibrium size distribution at high RH of the particles with well defined dry size was measured. The RH and temperature of the sheath air of the second DMA was monitored by a dew point mirror (EdgeTech DewMaster), while the RH and temperature of the sample flow at the inlet and after the humidifier were recorded by two additional sensors (Vaisala Humidity and Temperature Probe HMP333 at the inlet and HMP110 after the humidifier). The temperature of the second DMA and the dewpoint temperature were used to determine the RH inside the second DMA. Due to a cooler temperature environment, the RH in the second DMA was always slightly higher than after the humidifier which is important for a precise determination of the particle’s DRH. The dew point mirror used has a temperature uncertainty of ±0.15 °C which translates to an uncertainty of ±1.2% RH at RH=90% (ref. 51). When used in this configuration the hydration branch of the aerosol hysteresis curve was determined. The measurements were inverted using the inversion algorithm and toolkit (TDMAinv)^52^.

The HTDMA measurements of aerosol samples exhibiting uniform growth provides the hygroscopic growth factor in terms of mobility diameter, 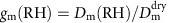, from which the volume-equivalent growth factor *g*_e_(RH) can be inferred:


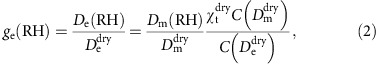


where *D*_e_(RH) is the volume-equivalent particle diameter at elevated RH and 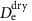 its corresponding dry value. 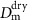 and *D*_m_(RH) are the mobility diameters at dry and elevated RH conditions, respectively. *C* represents the Cunningham slip correction. 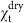 denotes the dynamic shape factor which is applied to the dry measurements only, since the solution droplets are spherical and no shape correction is needed (*D*_m_(RH)=*D*_e_(RH)).

The accuracy of the HTDMA was verified with measurements using ammonium sulfate. The recorded humidograms and DRH of ammonium sulfate were in agreement with theoretical expectations^1^,^22^. An overall measurement uncertainty of 5.5% (upper limit for all three HTDMA experiments) was estimated by combining, in quadrature, the instrumental uncertainty of the HTDMA (max. 4.7%) with the uncertainty of the measured shape-factor corrections (max. 2%, from the s.d. of repeated measurements).

### Hygroscopicity method II

As a second method we have measured the bulk solution water activity. To do so, we employed a chilled mirror instrument (AquaLab water activity metre, Model 3B, Decagon Devices, USA) to measure the dew point temperature of the gas phase in equilibrium with a bulk sample. The instrument was calibrated using saturated NaCl solution and distilled water as references. From dew point temperature and sample temperature the instrument provides water activity, *a*_*w*_, with a specified accuracy of 0.3%. Sea salt solutions were made using MilliQ water (resistivity ≥18.2 MΩ cm) and the same commercial sea salt mixture used to generate the sea salt particles. The estimated error in the solution preparation was ±0.005 in mass fraction. Solutions were allowed to equilibrate for at least one week before the measurements.

### Hygroscopicity method III

We have used a double ring EDB, which has been described in detail previously^53^,^54^. Temperature is controlled using a circulating cooling liquid and RH is controlled by adjusting a continuous flow of dry and humidified nitrogen gas using mass flow controllers. Single particles are injected into the balance using an ink jet cartridge (Hewlett Packard 51633A) and are inductively charged. Mass change of the levitated particle is measured using a feedback loop adjusting the DC-voltage compensating the gravitational force acting on the particle. RH is measured by a capacitive probe with an integrated temperature sensor (U.P.S.I., France, model G-TUS.13R) mounted in the upper-end cap of the EDB in close proximity to the levitated particle (<10 mm distance). Above ∼90% RH the sensor shows considerable hysteresis between humidifying and drying cycles and its accuracy is limited. Based on deliquescence measurements of different salts we estimate its accuracy below 90% RH to be ±1.5% RH.

To measure hygroscopic mass growth, we changed RH slowly (typically ∼28 h from dry to 95% RH and again ∼28 h for drying) to stay close to thermodynamic equilibrium. The hygroscopic mass growth factor, 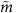, is defined as the mass at elevated RH divided by its dry value. To convert mass growth to size growth we use the dry density as measured by the helium-pycnometre (*ρ*_dry_=2.017 g cm^−3^), while we use the RH-dependent density parameterization of Tang *et al*.^10^ for seawater. For NaCl we use *ρ*_dry_=2.16 g cm^−3^ and the parameterization for the RH-dependent density of NaCl by Tang *et al*.^10^. In addition, we test the assumption that the volumes of solutes and water are additive when calculating the RH-dependent density^55^ (see Table 1) and show the difference to the parameterization of Tang *et al*.^10^ as error bars (see Figs 2c and 3).

### Thermodynamic modelling of inorganic sea salt hygroscopicity

We have used two thermodynamical models to calculate the hygroscopic growth of the sea salt using the measured size-dependent chemical composition as input. The chemical composition of the aqueous solutions were derived from measurements of the aerosol inorganic composition during artificial seawater experiments with the sea spray chamber used during this study^24^. Hygroscopic growth factors were determined from the same chemical composition as that used to derive the liquid phase water activity using the known mass fraction of the inorganic ions and assuming a density of 2.02 g cm^−3^ (see helium-pycnometre measurements above) for the dry sea salt. In addition, we used UManSysProp v1.0 (ref. 56) and the ADDEM model^22^ to calculate the hygroscopic growth of NaCl.

The first model used was the thermodynamic aerosol inorganics model (AIM^33^; see http://www.aim.env.uea.ac.uk/aim/aim.php) for the H^+^–NH_4_^+^–Na^+^–K^+^–Ca^2+^–Mg^2+^–SO_4_^2−^–NO_3_^−^–Cl^−^–CO_3_^2−^–OH^−^–H_2_O system (AIM-accent model). Since the calculations were performed using the (bulk) chemical composition measurements, the Kelvin effect was accounted for by using a modification of the parameterization of Lewis^23^ as follows: The parameterization is a first-order approximation of the size-dependent Kelvin effect derived from Köhler theory. At RH=90%, Lewis^23^ approximates a reduction in hygroscopic growth of −6 nm/*D*_dry_ due to the Kelvin effect which is only weakly dependent on solute properties. We have slightly adjusted this parameterization after comparing the original parameterization to the hygroscopic growth of NaCl predicted by the thermodynamical model of Topping *et al*.^22^. Thus, for NaCl we use a decrease in hygroscopic growth of −4.71 nm/*D*_m,dry_ at RH=90% (Supplementary Fig. 6).

The second model used was ISORROPIA-II (ref. 57), which is a code that treats the thermodynamics of K^+^–Ca^2+^–Mg^2+^–NH_4_^+^–Na^+^–SO_4_^2−^–NO_3_^−^–Cl^−^–H_2_O aerosol systems. ISORROPIA-II is designed to solve forward problems, in which known quantities are T, RH and the total (gas and aerosol) concentrations of NH_3_, H_2_SO_4_, Na^+^, HCl, HNO_3_, Ca^2+^, K^+^ and Mg^2+^, and, reverse problems, in which known quantities are T, RH and the concentrations of aerosol NH_4_^+^, SO_4_^2−^, Na^+^, Cl^−^, NO_3_^−^, Ca^2+^, K^+^ and Mg^2+^. The output of both problems is the concentration of species in gas and aerosol (solid/liquid) phase. ISORROPIA-II can predict composition for the stable (or deliquescent path) solution where salts precipitate once the aqueous phase becomes saturated with respect to a salt, and, a metastable solution, in which the aerosol is composed only of an aqueous phase regardless of its saturation state (except for CaSO_4_, which is assumed to precipitate spontaneously). For the data set of this study, the forward metastable mode of ISORROPIA-II is used to emulate the generation and gas-particle equilibration of aerosol generated in this study. ISORROPIA-II has been evaluated for its ability to predict water uptake, acidity and semivolatile partitioning of inorganic species in a number of studies^58^.

### Global aerosol model simulations

We have used a general circulation model (ECHAM-HAMMOZ; Version echam 6.1-ham2.2-moz0.9) here referred to as ECHAM6-HAM2 to investigate the implications of our measurements. The aerosol-climate model part, ECHAM6-HAM2, consists of the most recent version of the aerosol module HAM2 (refs 32, 59) which is coupled to the atmospheric general circulation model ECHAM6 (ref. 60). It solves the prognostic equations for vorticity, divergence, surface pressure and temperature. HAM2 uses the two-moment M7 aerosol microphysics scheme^61^ and a two-moment cloud microphysics scheme that includes prognostic equations for the cloud droplet and ice crystal number concentrations as well as cloud water and cloud ice^62^,^63^. The activation of aerosol particles into cloud droplets is parameterization by Barahona *et al*.^64^. Two independent sea spray source functions were used in ECHAM^34^,^35^.

The aerosol module, HAM2, calculates the global evolution of five aerosol species: sulfate, particulate organic matter, black carbon, sea salt and dust. These species are the constituents of both internally and externally mixed aerosol particles whose size distribution is represented by seven uni-modal log-normal distributions. These seven modes describe four size classes (nucleation, Aitken, accumulation and coarse) and two hygroscopic classes (hydrophobic and hydrophilic). The 5-year simulations (2006–2010) were performed at 1.9° × 1.9° spectral resolution using 31 vertical levels. The model was run in nudged configurations with emissions as described in a recent AeroCom study^65^. To test the impact of our measurement results, we have adjusted the hygroscopicity parameter *κ* (ref. 30) of the inorganic sea salt component (as represented by NaCl in the model) to the values determined in this study. We use *κ*_s_ to specifically denote the sea salt component.

### Code availability

The ECHAM6-HAMMOZ model is made available to the scientific community under the HAMMOZ Software Licence Agreement, which defines the conditions under which the model can be used. More information can be found at https://redmine.hammoz.ethz.ch/projects/hammoz/wiki/1_Licencing_conditions.

### Data availability

The data of this study are available on request from the corresponding author (P.Z.).

## Additional information

**How to cite this article:** Zieger, P. *et al*. Revising the hygroscopicity of inorganic sea salt particles. *Nat. Commun.* **8**, 15883 doi: 10.1038/ncomms15883 (2017).

**Publisher’s note:** Springer Nature remains neutral with regard to jurisdictional claims in published maps and institutional affiliations.

## Supplementary Material

Supplementary Information Supplementary Figures, Supplementary Tables and Supplementary References

Peer Review File

## Figures and Tables

**Table 1:** Summary of measured hygroscopic growth for inorganic sea salt particles and NaCl.

**Measurement**	***D***_**dry**_	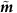 **(RH=90%)**	***g***_**e**_**(RH=90%)**	***κ***_**s**_**(RH=90%)**
Sea salt (HTDMA, chamber)	50 nm	—	2.12	1.14
Sea salt (HTDMA, chamber)	100 nm	—	2.12	1.04
Sea salt (HTDMA, chamber)	150 nm	—	2.09	0.96
Sea salt (HTDMA, nebulizer)	50 nm	—	2.22	1.31
Sea salt (HTDMA, nebulizer)	100 nm	—	2.20	1.17
Sea salt (HTDMA, nebulizer)	150 nm	—	2.19	1.12
Sea salt (EDB)	∼7 μm	5.24	2.09 (2.11)	0.91 (0.95)
Sea salt (water activity)	Bulk	5.83	2.18 (2.20)	1.04 (1.08)
NaCl (HTDMA, nebulizer)	50 nm	—	2.26	1.39
NaCl (HTDMA, nebulizer)	100 nm	—	2.29	1.34
NaCl (HTDMA, nebulizer)	150 nm	—	2.29	1.30
NaCl (EDB)	∼7 μm	6.1	2.29	1.22
NaCl (theory)	50 nm	6.40	2.33	1.51
NaCl (theory)	100 nm	6.73	2.38	1.49
NaCl (theory)	150 nm	6.85	2.39	1.48
NaCl (theory)	7 μm	7.10	2.42	1.47

Measured hygroscopic growth factors *g*_e_(RH), mass growth factors 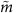
(RH=90%) and corresponding *κ*_s_ values of the inorganic sea salt at RH=90%. The values for *g*_e_(RH) of the EDB and the water activity measurements are calculated using the measured dry density of 2.017 g cm^−3^ and the RH-dependent density parameterization of Tang *et al*.^10^ for seawater. The values in parenthesis are calculated assuming that the volume of solutes and water are additive. The theoretical values for NaCl are shown as a reference (taken from ref. 56). The *κ*_s_ values were calculated at RH=90% and room temperature (*T*=298.15 K) according to Petters and Kreidenweis^30^ assuming the surface tension of water.
