# Supplementary material for: Revising the hygroscopicity of inorganic sea salt particles
Source: Nat Commun. 2017 Jul 3;8:15883. doi: 10.1038/ncomms15883 (PMC5500848; doi:10.1038/ncomms15883)
Supplement: Supplementary Information — Supplementary Figures, Supplementary Tables and Supplementary References [file ncomms15883-s1.pdf]

Title of file for HTML: Supplementary Information
Description: Supplementary Figures, Supplementary Tables and Supplementary References

Title of file for HTML: Peer Review File
Description:

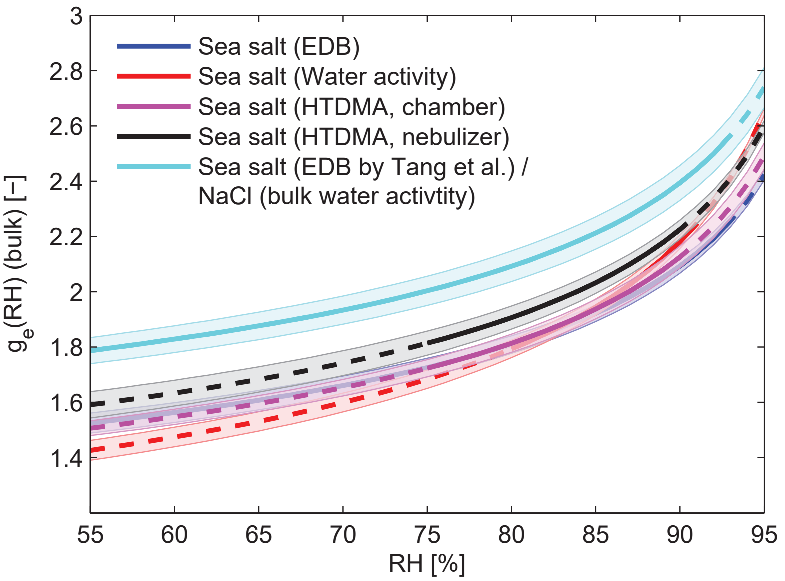

Supplementary Figure 1. Humidogram of the bulk hygroscopic growth factor for inorganic sea salt particles. Bulk hygroscopic growth factor g_e(RH) vs. relative humidity (RH) for sea salt particles determined from EDB, water activity and HTDMA measurements. Shaded areas for the EDB and water activity measurements give the absolute difference between the RH-dependent density parametrisation of Tang *et al.*[1] and using the assumption that the volumes of solutes and water are additive. The shaded areas for the HTDMA state the variation of the measurements at the three distinct dry diameters after the values were back-calculated to bulk values. Dashed lines indicate RH-ranges where the data were extrapolated. The ratio of g_e(RH) to the values of NaCl can be found in Fig. 2c in the main manuscript.

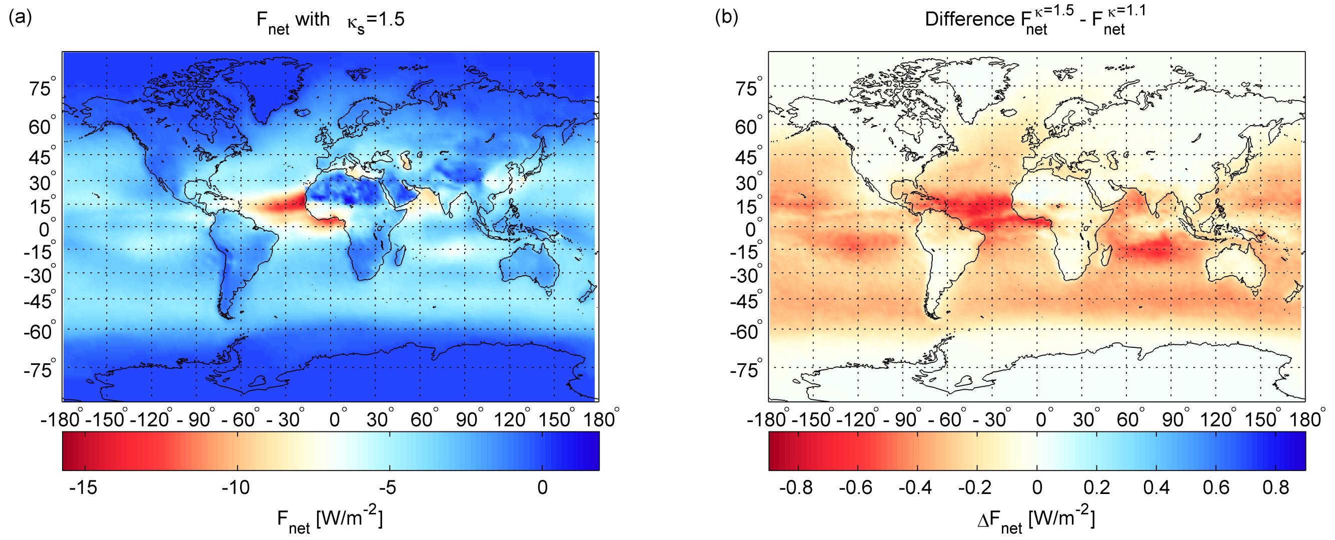

Supplementary Figure 2. **Global net radiative forcing.** **(a)** Clear sky and top of the atmosphere aerosol radiative forcing F_{net} (net value as sum of short- and longwave forcing) with the hygroscopic growth of the inorganic sea spray component set to $\kappa_s = 1.5$ (NaCl). **(b)** The absolute difference between F_{net} with $\kappa_s = 1.5$ and $\kappa_s = 1.1$. The global mean values are -3.36 Wm^2 and -3.14 Wm^2 for $\kappa_s = 1.5$ and $\kappa_s = 1.1$, respectively. Results are shown using the sea spray source function of Gong *et al.*[2].

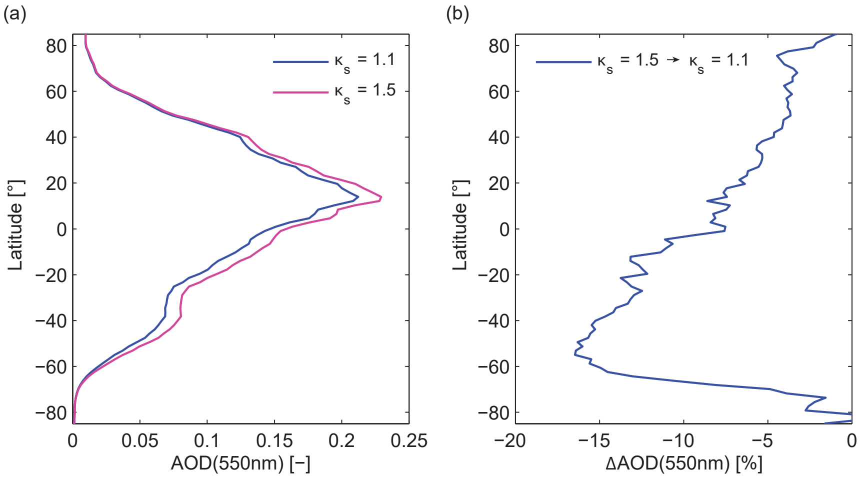

Supplementary Figure 3. Latitudinal mean of aerosol optical depth (AOD) using the sea spray source function of Long *et al.*. **(a)** Latitudinal mean of the AOD(550 nm) for $\kappa_s = 1.5$ and 1.1. **(b)** Percental change in AOD when decreasing the hygroscopic growth of the inorganic sea spray component from 1.5 to 1.1. Same as Fig. 4b and 4c (main manuscript) except that the sea salt source parametrisation of Long *et al.*[3] instead of Gong *et al.*[2] has been used.

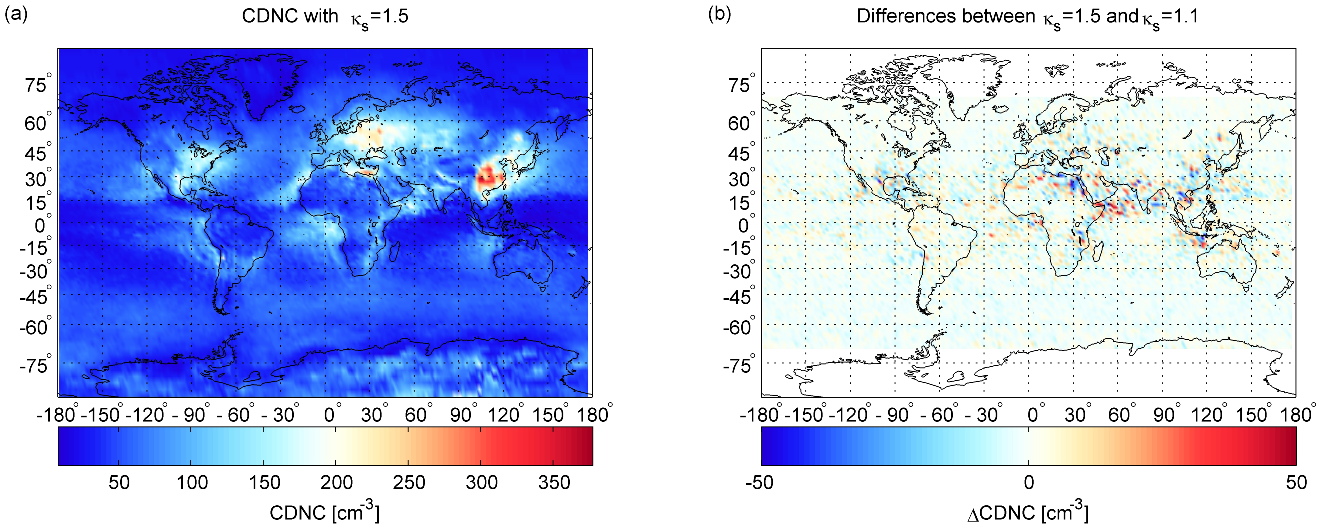

Supplementary Figure 4. Global cloud droplet number concentration. (a) The cloud droplet number concentration (CDNC) with the hygroscopic growth of the inorganic sea spray component set to $\kappa_s = 1.5$ (NaCl). **(b)** The absolute difference between CDNC with $\kappa_s = 1.5$ and $\kappa_s = 1.1$. The global mean values are $60.5\,\mathrm{cm}^{-3}$ and $60.9\,\mathrm{cm}^{-3}$ for $\kappa_s = 1.5$ and $\kappa_s = 1.1$, respectively. Results are shown using the sea spray source function of Gong *et al.*[2].

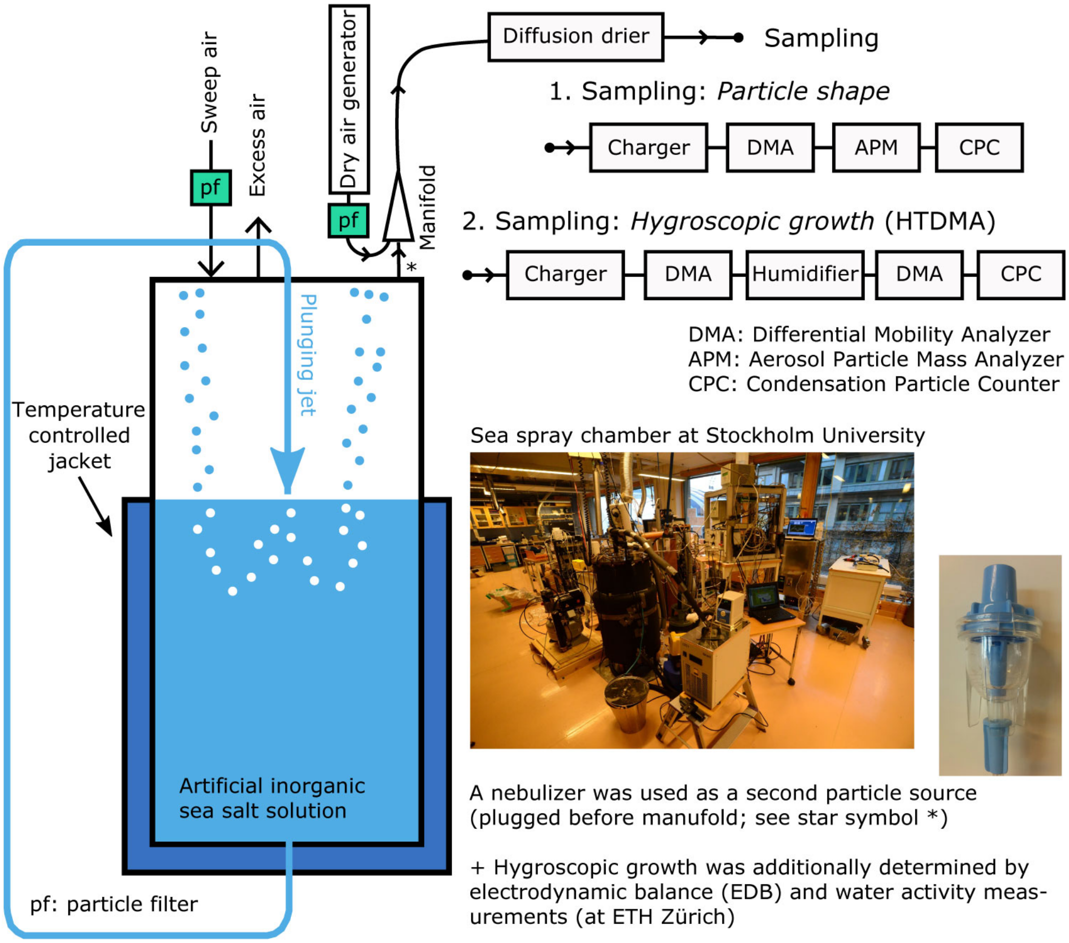

Supplementary Figure 5. Schematic setup of the sea spray chamber to produce nascent sea salt particles. The right hand side shows the setup used to measure the particle's dynamic shape factor and hygroscopic growth. A photo of the sea spray chamber and the nebulizer used as particle source are shown below. More technical details can be found in Salter *et al.*[4].

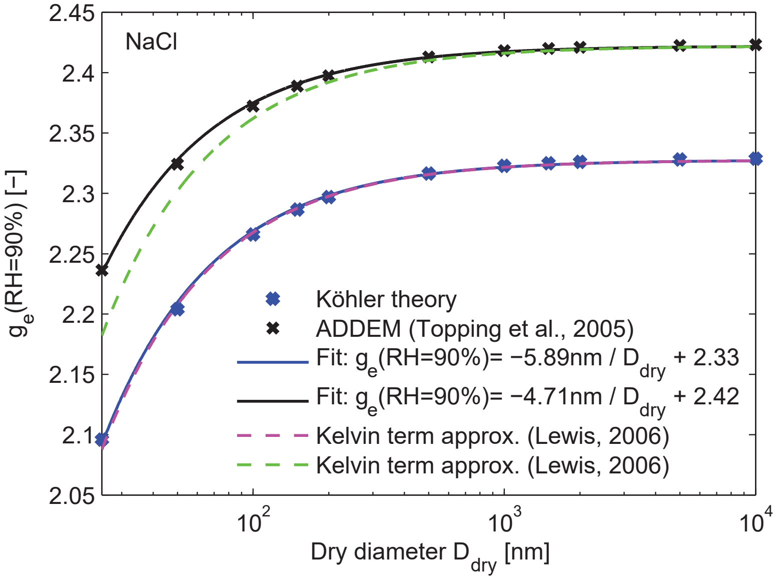

Supplementary Figure 6. **Modelled hygroscopic growth of NaCl.** Modelled hygroscopic growth factor $g_e(\mathrm{RH} = 90\%)$ vs. dry diameter for NaCl determined from Köhler theory (approximation for dilute droplets) and by the thermodynamical model ADDEM[5].T he solid lines represent fits using the equation $g(\mathrm{RH} = 90\%, D_{\mathrm{dry}}) = a/D_{\mathrm{dry}} + g_0$ (see legend for fit-coefficients). The dashed lines show the result of the Kelvin term parametrisation by Lewis[6] which uses the same fit-equation and $a = -6$ nm as a coefficient. We have used a modified version of the Lewis[5] parameterization where we use the factor $a = -4.71$ nm instead of the original value of $a = -6$ nm. The Lewis[6] parametrisation perfectly describes the hygroscopic growth using Köhler theory for a dilute solution droplet. The differences in comparison to the ADDEM model result from the fact that this model includes non-ideality effects.

Supplementary Table 1. Measurements and model results of the hygroscopic growth of NaCl and inorganic sea salt particles derived from HTDMA (hygroscopic tandem differential mobility analyser), water activity and EDB (electrodynamic balance) measurements.

	System	Aerosol generation[g]	Technique	g_e(RH)	D_{dry}	RH_{wet}	RH_{dry}	Shape factor	Reference
Lab studies	NaCl	N	HTDMA	2.07 (2.30)[l]	100 nm	85 %	< 5 %	?[i]	Koehler *et al.*[7]
	NaCl	N	HTDMA	2.46	71-77 nm	90 %	?[h]	$\chi = 1.21$-1.120[c]	Modini *et al.*[8]
	NaCl	N	HTDMA	2.4	100 nm	90 %	< 5 %	?	Laskina *et al.*[9]
	NaCl	N	HTDMA	2.40	100 nm	90 %	< 10 %	$\chi = 1.08$[f]	Fuentes *et al.*[10]
	NaCl	N	HTDMA	2.29	100 nm	90 %	< 20 %	$\chi = 1.07$[f]	Gysel *et al.*[11]
	**NaCl**	**N**	**HTDMA**	**2.28**[k]	**100 nm**	**90 %**	**8 %**	$\chi = 1.08$[e]	**This study**
	NaCl	?	EDB	2.27[k]	-	90 %	-	-	Tang[12]
	**NaCl**	**P**	**EDB**	**2.10 (2.29)**[k]	**$\sim 5\,\mu$m**	**85 %**	**<3%**	**-**	**This study**
	NaCl	-	Water activity	2.42[k]	bulk	90 %	-	-	Zamora *et al.*[13]
	**NaCl**	-	**Water activity**	**2.01(2.34)**[l]	**bulk**	**80 %**	**-**	**-**	**This study**
	Marine chloride mixture	N	HTDMA	2.1	100 nm	90 %	< 5 %	?	Laskina *et al.*[9]
	Artificial seawater[a]	F	HTDMA	2.35	71-77 nm	90 %	?	$\chi = 1.21$-1.120[c]	Modini *et al.*[8]
	Artificial seawater[b]	A	HTDMA	2.41	100 nm	90 %	< 10 %	$\chi = 1.08$[f]	Fuentes *et al.*[10]
	Artificial seawater[b]	J	HTDMA	2.46[k]	100 nm	90 %	< 10 %	$\chi = 1.08$[f]	Fuentes *et al.*[10]
	Artificial seawater[b]	F	HTDMA	2.46[k]	100 nm	90 %	< 10 %	$\chi = 1.08$[f]	Fuentes *et al.*[10]
	Artificial seawater[b]	D	HTDMA	2.46[k]	100 nm	90 %	< 10 %	$\chi = 1.08$[f]	Fuentes *et al.*[10]
	**Artificial seawater**[d]	**N**	**HTDMA**	**2.24**[k]	**100 nm**	**90 %**	**8 %**	$\chi = 1.14$[e]	**This study**
	**Artificial seawater**[d]	**J**	**HTDMA**	**2.11**[k]	**100 nm**	**90 %**	**5 %**	$\chi = 1.07$[e]	**This study**
	**Artificial seawater**[d]	**I**	**EDB**	**2.04-2.09**[m] **(2.19-2.25)**[m,k]	**$\sim 7\,\mu$m**	**85 %**	**<3 %**	-	**This study**
	**Artificial seawater**[d]	**-**	**Water activity**	**2.27-2.33**[m,k]	**bulk**	**90 %**	**-**	**-**	**This study**
	Natural seawater	F	HTDMA	2.26	71-77 nm	90 %	?	$\chi = 1.21$-1.120[c]	Modini *et al.*[8]
Model studies	NaCl	-	UNIFAC	2.44	bulk	90 %	0 %	-	Ming and Russell[14]
	NaCl	-	ADDEM	2.37	100 nm	90 %	0 %	-	Topping *et al.*[5]
	Inorganic seawater	-	UNIFAC	2.27	bulk	90 %	0 %	-	Ming and Russell[14]
	**NaCl**	**-**	**E-AIM**	**2.42**	**bulk**	**90 %**	**0 %**	-	**This study**
	**Inorganic seawater**	**-**	**E-AIM**	**2.33**	**bulk**[n]	**90 %**	**0 %**	**-**	**This study**
Field studies	Sea salt	Ambient aerosol	HTDMA	~ 2.1	100 nm	90 %	?	?	Swietlicki *et al.*[15]

[a] Seinfeld and Panids[16]; Niedermeier *et al.*[17]
[b] Kester *et al.*[18]
[c] Biskos *et al.*[19]
[d] See main text for details.
[e] Measured χ.
[f] Here the authors assume perfect cubes but incorrectly use the χ value from the continuum regime rather than the transition regime. Transition regime $\chi = 1.20$ for perfect cubes with a diameter of 100 nm..
[g] N = nebulizer; F = glass frit; J = plunging jet; D = aquarium diffuser; I = Ink jet; ? = not specified.
[h] ? = not specified.
[i] Although this data was shape corrected no value for χ was specified.
[j] Mass growth factors were converted to g(RH) assuming a dry and wet density of NaCl. Later one was calculated using E-AIM.
[k] Interpolated to RH=90 %.
[l] Extrapolated to RH=90 %.
[m] Range shows values for two different dry densities in order to convert from mass to diameter growth factors.
[n] Chemical composition of last LPI filter stage no. 13 ($D_{mob} > 10.15\mu$m) see Salter *et al.*[20].

Supplementary Table 2. Parametrisation of the shape factor measurements. Fit-coefficients of the exponential decaying fit ($\chi_\mathrm{t}(D_\mathrm{m}) = a\exp(bD_\mathrm{m}) + c\exp(dD_\mathrm{m})$) applied to the measured shape factors χ_t as a function of mobility diameter D_m (see Fig. 1 in main manuscript). The values in brackets give the 95 % confidence bounds.

Param.	Sea salt (chamber)	Sea salt (neb.)	NaCl (neb.)
a	0.074 (0.031, 0.178)	0.32 (-0.56, 1.21)	0.109 (0.049, 0.170)
b	-0.0065 (-0.0214, 0.0084)	-0.0029 (-0.0104, 0.0045)	-0.0069 (-0.016, 0.0019)
c	1.034 (0.992, 1.077)	0.88 (-0.03, 1.79)	1.02 (0.98, 1.06)
d	-2.28e-05 (-6.07e-5, 1.52e-5)	0.00022 (-0.00087, 0.00131)	4.49e-05 (5.72e-6, 8.42e-5)

Supplementary References

1. Tang, I. N., Tridico, A. & Fung, K. Thermodynamic and optical properties of sea salt aerosols. *J. Geophys. Res.* **102**, 23269–23275 (1997).
2. Gong, S. A parameterization of sea-salt aerosol source function for sub-and super-micron particles. *Global Biogeochem. Cycles* **17**, 1097 (2003).
3. Long, M. S., Keene, W. C., Kieber, D. J., Erickson, D. J. & Maring, H. A sea-state based source function for size- and composition-resolved marine aerosol production. *Atmos. Chem. Phys.* **11**, 1203–1216 (2011). URL http://www.atmos-chem-phys.net/11/1203/2011/.
4. Salter, M. E., Nilsson, E. D., Butcher, A. & Bilde, M. On the seawater temperature dependence of the sea spray aerosol generated by a continuous plunging jet. *J. Geophys. Res.* **119**, 9052–9072 (2014). URL http://dx.doi.org/10.1002/2013JD021376.
5. Topping, D. O., McFiggans, G. B. & Coe, H. A curved multi-component aerosol hygroscopicity model framework: Part 1: Inorganic compounds. *Atmos. Chem. Phys.* **5**, 1205–1222 (2005). URL http://www.atmos-chem-phys.net/5/1205/2005/.
6. Lewis, E. R. The effect of surface tension (Kelvin effect) on the equilibrium radius of a hygroscopic aqueous aerosol particle. *J. Aerosol. Sci.* **37**, 1605–1617 (2006).
7. Koehler, K. A. *et al.* Water activity and activation diameters from hygroscopicity data - Part II: Application to organic species. *Atmos. Chem. Phys.* **6**, 795–809 (2006). URL http://www.atmos-chem-phys.net/6/795/2006/.
8. Modini, R. L., Harris, B. & Ristovski, Z. D. The organic fraction of bubble-generated, accumulation mode Sea Spray Aerosol (SSA). *Atmos. Chem. Phys.* **10**, 2867–2877 (2010). URL http://www.atmos-chem-phys.net/10/2867/2010/.
9. Laskina, O. *et al.* Size matters in the water uptake and hygroscopic growth of atmospherically relevant multicomponent aerosol particles. *J. Phys. Chem.* **119**, 4489–4497 (2015).
10. Fuentes, E., Coe, H., Green, D., Leeuw, G. d. & McFiggans, G. Laboratory-generated primary marine aerosol via bubble-bursting and atomization. *Atmos. Meas. Tech.* **3**, 141–162 (2010).
11. Gysel, M., Weingartner, E. & Baltensperger, U. Hygroscopicity of aerosol particles at low temperatures. 2. Theoretical and experimental hygroscopic properties of laboratory generated aerosols. *Environ. Sci. Technol.* **36**, 63–68 (2002).
12. Tang, I. Chemical and size effects of hygroscopic aerosols on light scattering coefficients. *J. Geophys. Res.* **101**, 19245–19250 (1996).
13. Zamora, I. R., Tabazadeh, A., Golden, D. M. & Jacobson, M. Z. Hygroscopic growth of common organic aerosol solutes, including humic substances, as derived from water activity measurements. *J. Geophys. Res.* **116** (2011).
14. Ming, Y. & Russell, L. Predicted hygroscopic growth of sea salt aerosol. *J. Geophys. Res.* **106**, 28259–28274 (2001).
15. Swietlicki, E. *et al.* Hygroscopic properties of submicrometer atmospheric aerosol particles measured with H-TDMA instruments in various environments - a review. *Tellus B* **60**, 432–469 (2008).
16. Seinfeld, J. & Pandis, S. *Atmospheric Chemistry and Physics: From Air Pollution to Climate Change* (John Wiley and Sons, Inc., Hoboken, New Jersey, 2006).
17. Niedermeier, D. *et al.* LACIS-measurements and parameterization of sea-salt particle hygroscopic growth and activation. *Atmos. Chem. Phys.* **8**, 579–590 (2008).
18. Kester, D. R., Duedall, I. W., Connors, D. N. & Pytkowicz, R. M. Preparation of artificial seawater. *Limnol. Oceanogr.* **12**, 176–179 (1967).
19. Biskos, G., Russell, L., Buseck, P. & Martin, S. Nanosize effect on the hygroscopic growth factor of aerosol particles. *Geophys. Res. Lett.* **33** (2006).
20. Salter, M. E. *et al.* Calcium enrichment in sea spray aerosol particles. *Geophys. Res. Lett.* **43**, 8277–8285 (2016). URL http://dx.doi.org/10.1002/2016GL070275. 2016GL070275.
