# Supplementary material for: Revising the hygroscopicity of inorganic sea salt particles
Source: Nat Commun. 2017 Jul 3;8:15883. doi: 10.1038/ncomms15883 (PMC5500848; doi:10.1038/ncomms15883)
Supplement: Peer Review File [file ncomms15883-s2.pdf]

Reviewers' comments:

Reviewer #1 (Remarks to the Author):

Review of NCOMMS-16-24262, “Revising the hygroscopicity of inorganic sea salt particles” by P. Zieger et al., submitted to Nature Communications.

The manuscript discusses experimental and modeling work pertaining to the hygroscopic growth of inorganic sea salt particles, with the basic conclusion being that the dependence of water uptake on relative humidity is weaker than the relationship typically used today, which is that reported by Tang and Munkelwitz (1993) based on measurements in an electrodynamic balance. The article is well written and covers an important topic that is of interest to the community. The work is comprehensive and the authors have used multiple approaches to arrive at their results. I would recommend that it be published, although additional discussion of some items is first required. That said, I am not convinced that the findings are of sufficient importance to justify publication in Nature Communications.

An issue that must be discussed relates to how sea salt particles contain associated water even when “dry.” The ability of sea salt, and artificial sea salt, to retain water, even when dried to low relative humidity (RH), is due to both magnesium and calcium forming stable compounds with water. This property is well known, and in the early 1900s provided a difficulty with regard to the determination of salinity of ocean water samples. The approach recommended for many years was to heat the salt to ~600C for 24 hours or so. If the mass of a dry sea salt particle was calculated from the inorganic salts, but MgCl2 was in the form MgCl2x6H2O (the stable form), and CaCl2 was in the form CaCl2x10H2O (the stable form), then the associated mass of water (the mass of 6H2O being 4.5 times that of Mg, and that of 10H2O being 4.5 times that of Ca) would be 4.5 times that of the sum of the masses of Mg and Ca, which comprise 5% or the sea salt particle mass. This would result in an overestimate of ~20% for the mass of the “dry” sea salt, and will result in an incorrect determination of the hygroscopic growth factor, and an error in the hygroscopicity factor kappa. Consideration of these effects will also modify the density of the particle. The density of dry inorganic sea salt, with no associated water, is roughly 2.2 g/cm^3, roughly independent of the assumptions made (i.e., the compounds which make up the sea salt); this is the value assumed by the authors (line 247). However, with associated water taken into account, the density is considerably lower. Making a very simple model of sea salt as being composed of NaCl, MgCl2, and Na2SO4 yields a density of 2.2 g/cm^3, but this decreases to 2.00 g/cm^3 if MgCl2 is in the form MgCl2x6H2O, and to 1.79 g/cm^3 if in addition Na2SO4 is in the form Na2SO4x10H2O.

Thus, with this associated water taken into account, the uptake of water would be less than what it would be for a totally dry (i.e., no associated water whatsoever) particle of the same mass by roughly 20%, very similar to the amount the manuscript claims is the difference between the measured value and the one typically used.

The authors did not discuss in detail how they dried the particle, but I would assume that their so-called dry particles actually contained some water. I would assume that Tang’s particles would have also, so this can’t account for the discrepancy between these results. However, failure to take into account the associated water may have resulted in differences between measured values and those calculated with thermodynamic routines. I would like to see a discussion of these considerations, which are important for this topic.

The impact of an incorrect assumption for the hygroscopicity parameter of inorganic sea salt, as demonstrated by model runs and shown in Fig. 4, is not very large. The maximum impact, taken as the largest difference between modeled AOTs when kappa was reduced from 1.5 to 1.1 (the extreme case), was at most ~0.02, and in most cases was much less. Considering that the sea salt production flux is uncertain to factors of 2 to 10, and that even concentrations are not known to within tens to hundreds of percent, at a minimum, and that most of the contribution to AOT is

probably from sea salt particles with diameters greater than one micrometer, for which concentrations are even more uncertain, the ability of a model to accurately represent the AOT is in no way limited by the hygroscopicity. EHCAM treats sea salt in only two modes, with a separation at one micrometer diameter, and thus the choice of the diameter that is used in each mode plays a very large role in the calculated values. Additionally, implicit in the calculation is that there is a known dry mass of sea salt, and that the diameters (and thus light-scattering abilities) of the wet particles are determined from growth factors (or kappa values), but the mass of sea salt in the atmosphere is very poorly constrained. These statements are in no way meant to diminish the importance of this paper or to imply that it is not worth examining this topic, but merely that their use of a model to demonstrate the importance of the finding was not especially compelling.

The main conclusion of the manuscript was there is a large discrepancy in the hygroscopicity parameter for inorganic sea salt, and that the value 1.5 which is typically used should be replaced by 1.1. The value determined for sea salt particles generated with the nebulizer ranged from 1.2 to 1.4, with a mean of 1.3; this itself is roughly half of the discrepancy the manuscript suggests should be changed. The reasons for the large difference in the hygroscopicity of particles generated by the nebulizer and those generated in the chamber is not satisfactorily explained. The recommendation (line 202) of a bulk value for kappa ranging from 1.06 to 1.29 is a wide target. It seems difficult to justify a change in the value for submicrometer sea salt particles when even bulk values are so difficult to constrain. Some further discussion of why there was such a large difference between results from the nebulizer and the sea spray chamber seems necessary.

The comments below are minor, but may improve the readability of the manuscript.

Although the authors explicitly state that they are dealing with “inorganic sea salt particles”, as reflected in the title, they mention “sea spray particles” and “inorganic sea spray particles” several times in the text. Sea spray particles are generally considered those that contain organics in addition to sea salt. Thus perhaps restricting discussion only to “sea salt particles” or “inorganic sea salt particles” might be advisable to remove any possibility of confusion.

The term “hygroscopicity” is used throughout the document, but in different ways. The colloquial meaning (such as used on lines 25, 27, 30, 32, and elsewhere) refers to the ability of a particle to uptake water substance. However, throughout the manuscript this term was used in a more quantitative manner to refer to a numerical value. Perhaps it would be preferable to use the term in only one sense or the other.

On line 67 and thereafter, the authors stated that NaCl transitioned from almost cubical to nearly spherical, with smaller particles having more rounded edges. While this explanation might be consistent with a dynamical shape factor more near unity, it seems to be merely an assumption, and not a conclusion, as it was stated.

Artificial sea salt is well known to contain additives to reduce clumping, and sometimes organic substances also. Was the mixture baked before use to remove organics?

How was the dynamic shape factor for a perfect cube as a function of mobility diameter determined (Figure 1)? This value should asymptote to 1.08 for large Dmob, and should attain the value 1.24 for very small Dmob. Perhaps these could be shown on the figure.

Why is the ADDEM result expected to be higher (line 117)? There was no previous discussion of this model before this statement.

Reviewer #2 (Remarks to the Author):

Anonymous review of "Revising the hygroscopicity of inorganic sea salt particles" by Zieger et al.

This laboratory study investigates and revises the hygroscopicity of inorganic sea salt aerosol, an important parameter used in global modeling of aerosol effects on climate. It is detailed and well written and should be accepted to Nature Communications. The majority of my comments are minor, but I would like the authors to consider discussing the implications of their work in the context of internally mixed particles. Their laboratory work focuses on the inorganic component of sea salt particles, but how important is it also to consider the organic component? Would considering this component even further reduce hygroscopic growth and be important for climate models to consider? I would suggest to consider this in the discussion and impacts section.

Below I give only minor comments:
1. Abstract:
"The particle generation method is an important factor for hygroscopicity measurements since it determines the particle's shape and chemical composition. We report, for the first time, size-dependent hygroscopic growth for particles smaller than 150 nm in diameter. This observation is independent of the particle generation method, and likely caused by size-dependent changes in particle solubility or surface composition in the submicrometer particlerange. "

-- I found these sentences a bit confusing to read the first time since you first say particle generation method is important then seem to contradict this in the third sentence above. Consider rewording somehow to make your meaning more clear.

2. Avoid using "state-of-the-art" buzzword - I'm sure all models consider themselves so!

3. Fig 2. Consider plotting full theoretical curve for NaCl (hydration and dehydration)

4. Line 117: Suggest a short sentence describing the models, or point to section where this occurs

5. Fig 4 and S4: Please add global mean on 2D plots; also, the notation of title is confusing as at first I thought Fnet was 1.5 W/m2 (not that K = 1.5).

6. In your discussion you imply inorganic part is most important (over organic) for reduced hygroscopicity, but you did not test this so how do you know?

7. Very interesting that the global model accidentally was using the "right" K. I wonder if other models did similarly? Maybe look at AeroCom results?

Reviewer #3 (Remarks to the Author):

Review report on the manuscript NCOMMS-16-24262 entitled "Revising the hygroscopicity of inorganic sea salt particles".

General comments:
This study is primarily based on measurements of the hygroscopicity of both sub-micrometer (humidified tandem differential mobility analyzer, HTDMA) and super-micrometer (electrodynamic balance, EDB) inorganic sea salt aerosol particles generated in laboratory from artificial inorganic sea salt solutions assumed as sea water by the authors. The study seems technically sound, but rather incremental than novel. The measurement methods used are well known to the community, but have been executed meticulously.

The major claim by the authors in this study is that “the measured reduction in the inorganic sea salt aerosol hygroscopicity, κ_s, from 1.5 (NaCl, considered as model sea salt) to 1.1 (NaCl with other salts of $MgCl_2$ and $CaCl_2$) manifested in a decrease of up to ~15% in aerosol optical depth (AOD), while indirect or cloud related effects were practically insensitive to this change”. The conclusions are limited by assumptions that sea spray aerosol (SSA) particles are composed of the inorganic sea water components only. On the other hand, several previous reports have proven beyond doubt that fresh sea-spray particles either collected from remote marine locations or generated in the presence of sea surface planktons in the laboratory or ocean-atmosphere facilities are dominated by organics at the submicron level1, 2, 3, 4, 5 . Furthermore, the inorganic components react within very short time leading to depletion of chloride and formation of nitrates and/or nss or non-sea-salt sulfates, etc.5, 6, 7, 8 . It is not clear how the results from this study can be used or extended towards the realistic parametrization (hygroscopicity and optical properties) of the more chemically complex sea-spray particles on a global scale and hence raises doubts about itsimpact on the wider atmospheric research community. The results, however, may provide some insight to researchers, specialized in the field, for future studies on the hygroscopicity of the inorganic components in real, ambient, marine aerosol species. I have my doubts whether the current version of the manuscript is publishable in nature-communications. I would suggest more specialized journals in this field. The authors need to address following major concerns in order to create greater impact.

Major Comments:

1. Continuing from the general comments above, the authors need to make it clear how the results from this study can be used or extended towards the parametrization (hygroscopicity and optical properties) of the real and more chemically complex sea-spray particles, which they claim are not easy to validate for a number of reasons as given in Pg. 2, lines 39-45.

2. How do the authors assume that the inorganic composition of the sea salt aerosol particles is primarily responsible for the suppression of the hygroscopic growth below NaCl rather than organic contamination (Pg. 4, lines 190-192)? Did the authors find comparable values of hygroscopicity parameter κ_s in literature (e.g. for Modini et al. in Table S1)? This and other such instances involving hygroscopicity of particles generated from natural sea-water should be highlighted and quantitatively stated in the main text.

3. The authors state that “if the surface composition is driving the water uptake at small sizes, only minute amounts of surface-tension-reducing substances are needed to result in a notable effect” (Pg.3, line 137). Which inorganic components (artificial sea water), with reduced surface tension, do the authors think can partition to the surface of the smallest particles and cause the observed increase in water uptake beyond thermodynamic predictions? How would organic contaminants, especially surface active organics and/or water insoluble organics, affect these small particles? Some clear arguments here can benefit the readers more than just cited literature.

4. The measured growth factors ge (RH =90 %) for pure NaCl particles have been plotted in Figure 3 and listed in Table S1. It would be better if authors include the hygroscopicity parameter κ_s values for NaCl calculated from these experimentally measured values in Table 1 along with the theoretical values. That should strengthen the authors’ claim that the particle generation methods are not responsible for the measured reduction in hygroscopicity of sea-salt particles compared to NaCl and showcase better reproducibility of the measurement conditions.

5. (Pg.5 lines 221-225) How do the authors ensure that particles are completely dry (5–8 % in HTDMA and < 3 % in EDB as listed in Table S1) given that sea-salt aerosols and $MgCl_2$ particles reportedly exhibited presence of residual water at RHs < 2 %9, 10, 11? Were the hydrationdehydration cycles repeated? Is there a possibility that the laboratory generated droplets when passed through diffusion dryer may exhibit kinetic artefacts (restructuring) due to fast drying12 . What could be the effect on shape factors when particles are dried through the diffusion dryer compared to slow equilibrated dehydration in the experimental loop?

6. Authors claim that "finally, the climate impact of our results are illustrated using a state-of-theart large-scale model" (Pg.2, Line 58). Plots of the AOD (Figure 4 and S5) estimated by running ECHAM6-HAM2 global climate model (GCM) considering reduced hygroscopicity parameter κs = 1.1 for sea salt particles (instead of 1.5) does show a significant reduction. How does the GCM obtained AODs compare with the real situation (at least some measured regional AODs, say in the Southern Ocean)? Authors should tone down their claims as the several studies on marine aerosol composition (cited in general comments) conducted post Tang et al13 clearly indicates that sea-spray aerosols are evidently more complex and NaCl or just pure sea-salt (NaCl with other salts of MgCl2 and CaCl2) may no longer be suitable representatives. A discussion may be included on whether GCMs need to be modified in order to accommodate different classes of sea spray aerosols with low uncertainty in hygroscopicity for each class. How does the constrained hygroscopicity parameter κs = 1.1 represent particles with organic contaminants which might have much lower hygroscopicity? How realistic is this applied constraint? If authors think that pure sea salt particles have an important role in the parametrization, relevant enough to the marine atmosphere, they should mention the abundance of such particles vis-a-vis other types containing organic and/or reacted species (as mentioned in general comments) citing literature.

Specific Comments:
1) (Pg.1, line 30). A sentence or two about the implications on heterogeneous chemistry due to the presence of condensed water over a wider RH range in sea salt aerosols compared to NaCl, citing literature14, 15 , may be added in the "discussion and impact" section.

2) (Pg.2, line 46) Please simplify the sentence for better clarity.

3) (Pg. 2, lines 77-79 and Pg.3, line 93-94) A mutual deliquescence of the MgCl2-dominant eutonic component in NaCl/MgCl2 mixed particles at RH = 15-16 % was reported in recent literature14 which can be cited here. The final DRH value due to the major salt, NaCl should be dependent on the mole fraction of NaCl in the salt mixture and hence should be slightly lower than that of pure NaCl (75.3 % at 25°C).

4) (Pg.3, lines99-100) Check the closing parenthesis "(although 3.5 % lower) than the……)".

5) (Pg.5, shape factor measurements) What is the percentage contribution of shape factor (χ) uncertainty in the ge and κs calculations? In Figure 1, the uncertainty in χt for nebulized smallest particles seems larger than that of large-mass particles. What could be the reason?

References

1. Facchini MC, et al. Primary submicron marine aerosol dominated by insoluble organic colloids and aggregates. Geophys Res Lett 35, L17814 (2008).

2. Quinn PK, et al. Contribution of sea surface carbon pool to organic matter enrichment in sea spray aerosol. Nature Geosci 7, 228-232 (2014).

3. Quinn PK, Collins DB, Grassian VH, Prather KA, Bates TS. Chemistry and Related Properties of Freshly Emitted Sea Spray Aerosol. Chem Rev, (2015).

4. Ault AP, et al. Size-Dependent Changes in Sea Spray Aerosol Composition and Properties with Different Seawater Conditions. Environ Sci Technol 47, 5603-5612 (2013).

5. Prather KA, et al. Bringing the ocean into the laboratory to probe the chemical complexity of sea spray aerosol. Proc Natl Acad Sci 110, 7550-7555 (2013).

6. Ault AP, et al. Heterogeneous Reactivity of Nitric Acid with Nascent Sea Spray Aerosol: Large Differences Observed between and within Individual Particles. J Phys Chem Lett 5, 2493-2500 (2014).

7. Ault AP, et al. Inside versus Outside: Ion Redistribution in Nitric Acid Reacted Sea Spray Aerosol Particles as Determined by Single Particle Analysis. J Am Chem Soc 135, 14528-14531 (2013).

8. Laskin A, et al. Reactions at Interfaces As a Source of Sulfate Formation in Sea-Salt Particles. Science 301, 340-344 (2003).

9. Tang IN, Tridico AC, Fung KH. Thermodynamic and optical properties of sea salt aerosols. J Geophys Res — Atmos 102, 23269-23275 (1997).

10. Cziczo DJ, Nowak JB, Hu JH, Abbatt JPD. Infrared spectroscopy of model tropospheric aerosols as a function of relative humidity: Observation of deliquescence and crystallization. J Geophys Res — Atmos 102, 18843-18850 (1997).

11. Cziczo DJ, Abbatt JPD. Infrared Observations of the Response of NaCl, MgCl2, NH4HSO4, and NH4NO3 Aerosols to Changes in Relative Humidity from 298 to 238 K. J Phys Chem A 104, 2038-2047 (2000).

12. Mikhailov E, Vlasenko S, Martin ST, Koop T, Pöschl U. Amorphous and crystalline aerosol particles interacting with water vapor: conceptual framework and experimental evidence for restructuring, phase transitions and kinetic limitations. Atmos Chem Phys 9, 9491-9522 (2009).

13. Tang IN. Thermodynamic and optical properties of mixed-salt aerosols of atmospheric importance. J Geophys Res — Atmos 102, 1883-1893 (1997).

14. Gupta D, Eom HJ, Cho HR, Ro CU. Hygroscopic behavior of NaCl–MgCl2 mixture particles as nascent sea-spray aerosol surrogates and observation of efflorescence during humidification. Atmos Chem Phys 15, 11273-11290 (2015).

15. Gupta D, Kim H, Park G, Li X, Eom HJ, Ro CU. Hygroscopic properties of NaCl and NaNO3 mixture particles as reacted inorganic sea-salt aerosol surrogates. Atmos Chem Phys 15, 3379-3393 (2015).

"Revising the hygroscopicity of inorganic sea salt particles" - Response to reviewer comments -

We would like to thank all three reviewers for their thorough and well-thought out reviews of our manuscript. The comments on the presence of hydrates were especially useful and have helped to improve our understanding. Indeed, accounting for these comments strengthened our paper since many previous publications on sea spray aerosol do not account for hydrates.

We would like to summarise the main conclusions of our work once more to facilitate the interpretation of this response letter:

1. The hygroscopicity of inorganic sea salt is significantly lower compared to NaCl. Therefore, hygroscopicity measurements of artificial sea salt should always be the baseline reference instead of pure NaCl.

2. Improving our understanding the behaviour of the inorganic fraction of sea spray is a prerequisite for understanding the hygroscopicity of the complex mixture that is sea spray aerosol, including the effect of organic substances.

3. Inorganic hydrates that are present even at low relative humidities are clearly important for sea spray hygroscopicity.

4. Our results have relevance beyond hygroscopicity - it is common practice to estimate the organic fraction of sea spray aerosol based on their volatility (see e.g. Modini et al., 2010). Since most of what these authors measure may be the decomposition of hydrates this suggests this approach may be invalid (see recent measurements of the volatility of sea spray aerosol by Rasmussen et al., 2017).

Resulting from the reviewer's comments, we have conducted the following additional experiments and model simulations to further consolidate these points:

1. We have confirmed experimentally, using Fourier transform infrared spectroscopy (FTIR) spectroscopy, that our inorganic sea salt mixture contains hydrates at conditions relevant for the atmosphere. Due to their presence, we have also determined the (dry) density of the salt mixture using a helium-pycnometer and re-analysed our experimental data based upon this density.

2. We have performed additional GCM simulations using a second sea spray source function to highlight that the effect of changing the sea salt hygroscopicity on the aerosol optical depth in relative terms is independent of the magnitude of sea salt emissions.

What follows is a point-by-point response to each of the points raised by the reviewers (black text).

1 Reviewer #1 (Remarks to the Author)

The manuscript discusses experimental and modeling work pertaining to the hygroscopic growth of inorganic sea salt particles, with the basic conclusion being that the dependence of water uptake on relative humidity is weaker than the relationship typically used today, which is that reported by Tang and Munkelwitz (1993) based on measurements in an electrodynamic balance. The article is well written and covers an important topic that is of interest to the community. The work is comprehensive and the authors have used multiple approaches to arrive at their results. I would recommend that it be published, although additional discussion of some items is first required. That said, I am not convinced that the findings are of sufficient importance to justify publication in Nature Communications.

An issue that must be discussed relates to how sea salt particles contain associated water even when "dry." The ability of sea salt, and artificial sea salt, to retain water, even when dried to low relative humidity (RH), is due to both magnesium and calcium forming stable compounds with water. This property is well known, and in the early 1900s provided a difficulty with regard to the determination of salinity of ocean water samples. The approach recommended for many years was to heat the salt to ~600C for 24 hours or so. If the mass of a dry sea salt particle was calculated from the inorganic salts, but MgCl2 was in the form MgCl2x6H2O (the stable form), and CaCl2 was in the form CaCl2x10H2O (the stable form), then the associated mass of water (the mass of 6H2O being 4.5 times that of Mg, and that of 10H2O being 4.5 times that of Ca) would be 4.5 times that of the sum of the masses of Mg and Ca, which comprise 5% or the sea

salt particle mass. This would result in an overestimate of ~20% for the mass of the "dry" sea salt, and will result in an incorrect determination of the hygroscopic growth factor, and an error in the hygroscopicity factor kappa. Consideration of these effects will also modify the density of the particle. The density of dry inorganic sea salt, with no associated water, is roughly 2.2 g/cm^3, roughly independent of the assumptions made (i.e., the compounds which make up the sea salt); this is the value assumed by the authors (line 247). However, with associated water taken into account, the density is considerably lower. Making a very simple model of sea salt as being composed of NaCl, MgCl2, and Na2SO4 yields a density of 2.2 g/cm^3, but this decreases to 2.00 g/cm^3 if MgCl2 is in the form MgCl2x6H2O, and to 1.79 g/cm^3 if in addition Na2SO4 is in the form Na2SO4x10H2O.

Thus, with this associated water taken into account, the uptake of water would be less than what it would be for a totally dry (i.e., no associated water whatsoever) particle of the same mass by roughly 20%, very similar to the amount the manuscript claims is the difference between the measured value and the one typically used.

The authors did not discuss in detail how they dried the particle, but I would assume that their so-called dry particles actually contained some water. I would assume that Tang's particles would have also, so this can't account for the discrepancy between these results. However, failure to take into account the associated water may have resulted in differences between measured values and those calculated with thermodynamic routines. I would like to see a discussion of these considerations, which are important for this topic.

Reply: We agree and thank the reviewer for this important comment. Indeed, this comment further highlights the importance of understanding the data presented in Tang et al. (1997) which led the same authors to conclude that the hygroscopicity of aerosol particles generated from natural seawater is almost identical to NaCl (the hygroscopicity of which is used to represent sea salt in many climate models). Further consideration of the data presented in Tang et al. (1997) (as well as further publications by the same authors) *suggests* that they have been corrected for the remaining water in the particles. In Tang et al. (1997) (Fig. 1), the dry aerosol mass is used as reference to plot the mass growth factor, while it states later in the manuscript (Sect. 3.1, last sentence) that *"some 5-10 wt% residual water is always found to be present in the solid particles"*. Therefore, it seems *likely* that the data presented by Tang et al. (1997) is actually the anhydrous dry mass (without any remaining water). However, we cannot be sure.

This is a critical point. If Tang et al. (1997) have corrected for the residual water, then many in the aerosol community have misinterpreted this data and the values have been transcripted as presented by Tang et al. (1997) into climate models. Further, if this is the case, then our results are in complete agreement with Tang et al. (1997) and both our study and that presented by Tang et al. (1997), suggest that sea salt aerosol particles have a significantly lower hygroscopicity than NaCl due to hydrates.

To elaborate upon this, we have determined the presence of hydrates in the sea salt we used by:

1. measuring the water content of the dry salt using FTIR spectrometry;
2. measuring the density of the dry inorganic sea salt.

The FTIR measurements clearly revealed the presence of water in the dry salt (see Fig. 1) which was not just physisorbed but crystallised within the structure of the salt crystals. Baking of the salt at 70 °C for three days did not cause any reduction in the amount of water present, suggesting that the hydrates present decompose at temperatures higher than this. We also attempted to bake the salt at temperatures above 100 °C. However, this visually changed the morphology of the salt. In addition, we believe that higher temperatures would not be atmospherically relevant anymore.

In the initial version of the manuscript, we assumed the density of NaCl was representative of the density of the inorganic sea salt mixture (which is dominated by NaCl). However, as the reviewer has correctly pointed out, the remaining water will cause a decrease in the actual density. Therefore, we have determined the density of the dry salt experimentally using a helium-pycnometer (AccuPyc 1330, Micromeritics Instrument Cooperation). This resulted in a measured density of $(2.017 \pm 0.006) \mathrm{g cm^{-3}}$ (mean ± standard deviation of 10 runs for three individual samples) which is significantly lower than the density we originally assumed. However, this is significantly higher than the density of $1.79 \mathrm{g cm^{-3}}$ estimated by the reviewer.

Our measurements of the dynamic shape factor constrain the lower limit of the sea salt density. If the density of the sea salt is lower than $\sim 2.0 \mathrm{g cm^{-3}}$ our calculated dynamic shape factors decrease to below 1 - values that are physically unreasonable.

As a result of these measurements we have used the new and directly measured density of $2.017 \mathrm{g cm^{-3}}$ throughout the manuscript and re-analysed our data. The decrease in density from that previously assumed has had a small effect on the measured dynamic shape factors (at maximum $< 5\%$ reduction in the shape factors) which, in turn, has influenced the HTDMA measurements of the hygroscopic growth (at maximum $< 3\%$ change in hygroscopic growth factors measured with the HTDMA). **However, these small changes do not change the overall conclusions of our paper.**

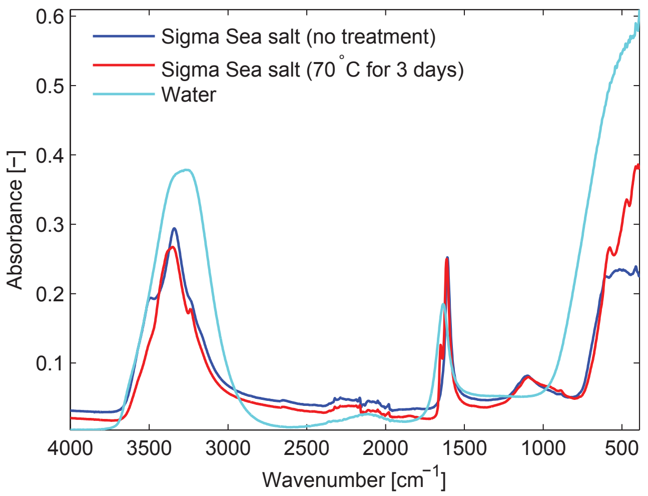

Figure 1: Infrared spectra (measured with a FTIR spectrometer Varian 670-IR) of the untreated Sigma sea salt (blue curve), the Sigma sea salt baked at 70°C for 72 hours (red curve) and for pure water (cyan curve). The contribution of water in the form of hydrates (water that is not just physisorbed but crystallised within the structure) is clearly visible.

Having said that, the importance of hydrates was not clear enough in the initial version of the manuscript. We have clarified the role of hydrates for our results by making the following changes:

We have added the following sentence to the methods section of the manuscript which includes the reference to the work of Cziczo et al. (1997):

"Even at very low RH, sea salt contains water in the form of hydrates[45]. We have confirmed this using Fourier transform infrared (FTIR) spectroscopy. The density of the artificial sea salt was experimentally determined using a helium-pycnometer (AccuPyc, Micromeritics Instrument Cooperation) and found to be $(2.017 \pm 0.006)\,\mathrm{g cm^{-3}}$ (mean ± standard deviation)."

This experimentally determined density is very close to the lower limit of $\rho = 2.0$ to retrieve reasonable shape factors (values of $\chi_t \geq 1$). We therefore added to the method section (shape factor measurements):
"For the sea salt particle we used the experimentally determined value of $\rho_s = 2.017\,\mathrm{g cm^{-3}}$ (see section above). It should be noted that $\rho_s = 2.0\,\mathrm{g cm^{-3}}$ is at the lower limit of density values to retrieve reasonable shape factors with χ_t generally ≥ 1."

We have added to the discussion and impact section the following paragraph:
"Inorganic sea salt contains water even at very low RH as an integral part of the crystalline structure. Examples of these hydrates are $MgCl_2 \cdot 6H_2O$ and $CaCl_2 \cdot 10H_2O$. These sea salt hydrates are present in all measurements of sea spray aerosol including those made by Tang *et al.*[10] who stated in their paper that 5-10 wt% water was always present in their particles at low RH. That hydrates are present in both the sea salt particles in the present study and in the measurements conducted by Tang *et al.*[10] means that they cannot explain the difference in the hygroscopic growth between the two studies. However, if Tang *et al.*[10] removed the contribution of water to the dry mass in their EDB measurements this may explain these differences. This observation is critical - if this residual water was removed by Tang *et al.*[10] then their values for the hygroscopic growth of sea salt (which would not include the atmospherically relevant hydrates) have been erroneously transcripted into atmospheric models. Notably, the presence of hydrates in sea salt under atmospherically relevant conditions also has relevance beyond hygroscopicity - it is common practice to estimate the organic fraction of sea spray aerosol based on the volatility of aerosols (see e.g. Modini *et al.*[21]). Since a significant fraction of what these authors measure is the decomposition of hydrates this suggests this approach should be used with caution (see recent work by Rasmussen et al.[40] on sea spray volatility)."

Comment: The impact of an incorrect assumption for the hygroscopicity parameter of inorganic sea salt, as demonstrated by model runs and shown in Fig. 4, is not very large. The maximum impact, taken as the largest difference between modeled AOTs when kappa was reduced from 1.5 to 1.1 (the extreme case), was at most ~0.02, and in most cases was much less. Considering that the sea salt production flux is uncertain to factors of 2 to 10, and that even concentrations are not known to within tens to hundreds of percent, at a minimum, and that most of the contribution to AOT is probably from sea salt particles with diameters greater than one micrometer, for which concentrations are even more uncertain, the ability of a model to accurately represent the AOT is in no way limited by the hygroscopicity. EHCAM treats sea salt in only two modes, with a separation at one micrometer diameter, and thus the choice of the diameter that is used in each mode plays a very large role in the calculated values. Additionally, implicit in the calculation is that

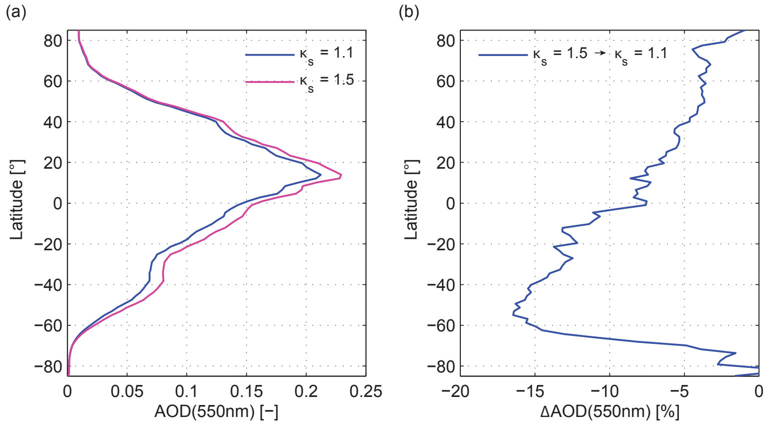

Figure 2: **(a)** Latitudinal mean of the AOD(550 nm) for κ_s = 1.5 and 1.1. **(b)** Percental change in AOD when decreasing the hygroscopic growth of the inorganic sea spray component from 1.5 to 1.1. Same as Fig. 4b and 4c in main manuscript except that the sea salt source parametrisation of Long et al. (2011) has been used.

there is a known dry mass of sea salt, and that the diameters (and thus light-scattering abilities) of the wet particles are determined from growth factors (or kappa values), but the mass of sea salt in the atmosphere is very poorly constrained. These statements are in no way meant to diminish the importance of this paper or to imply that it is not worth examining this topic, but merely that their use of a model to demonstrate the importance of the finding was not especially compelling.

Reply: We partially agree with the reviewer. The change in AOD between the model runs is locally significant with a reduction of up to 15 % (the net radiative forcing shows a similar decrease). Although, we are aware that many other model parameters (e.g., the sea spray source function estimates, removal parametrisations, uncertainties in meteorological fields, etc.) add to the uncertainty in AOD and other variables of interest, it is our view that as a community we need to keep improving atmospheric models piece by piece so as to improve the model performance in general.

By running ECHAM using two independent sea spray source functions (Gong, 2003; Long et al., 2011) we were able to test the impact of this uncertainty on our results (the difference between these two source functions can be seen in Fig. 5a in Salter et al. (2015)). The reduction in hygroscopicity that we observed (relative to NaCl) resulted in a relative change in AOD that was essentially independent of the sea spray source function (see Fig. 2, panel b).

To make this point clearer in the manuscript we have added the following sentence to the manuscript (and Fig. S6 to the supplement):

"We tested two independent sea spray source functions[63,64] and although the latitudinal average of the AOD was different depending on the source function used, the relative changes to AOD remained almost unchanged (see Supplementary Figure 6). A constrained hygroscopicity parameter for inorganic sea salt, as presented here, will therefore remove a systematic bias."

and in the method section:

"Two independent sea spray source functions were used in ECHAM[63,64]."

Comment: The main conclusion of the manuscript was there is a large discrepancy in the hygroscopicity parameter for inorganic sea salt, and that the value 1.5 which is typically used should be replaced by 1.1. The value determined for sea salt particles generated with the nebulizer ranged from 1.2 to 1.4, with a mean of 1.3; this itself is roughly half of the discrepancy the manuscript suggests should be changed. The reasons for the large difference in the hygroscopicity of particles generated by the nebulizer and those generated in the chamber is not satisfactorily explained. The recommendation (line 202) of a bulk value for kappa ranging from 1.06 to 1.29 is a wide target. It seems difficult to justify a change in the value for submicrometer sea salt particles when even bulk values are so difficult to constrain. Some further discussion of why there was such a large difference between results from the nebulizer and the sea spray chamber seems necessary.

Reply: It is our view that the aerosol generated by the sea spray chamber is a better proxy for natural sea spray aerosol than that produced by the nebulizer. This is the reason that we propose the lower value of κ should be used by the community. We are aware that there is a discrepancy between the nebulizer experiments and those conducted using the sea spray chamber and we discuss these openly in the manuscript See page 4, line 186 of the original manuscript: "With regards to the importance of the particle generation mechanism, we observe a clear difference in the hygroscopic growth and the dynamic shape factor of the particles generated with the plunging jet compared to the nebulizer. The reasons for this are unclear, but entraining air by impinging water from above, in a manner similar to the plunging jet deployed in our study, is

likely to be more representative of breaking waves than standard nebulizers.”). Unfortunately, we are unable to ascertain the exact mechanism and can only speculate that the composition of the particles produced by the nebulizer is different to those produced by the sea spray chamber.
We have added to this paragraph a clarification that the measurements of the chamber should be regarded as the most representative value:
”For this reason, the hygroscopic growth of the particles generated by the sea spray chamber are likely to better reflect the hygroscopic growth of nascent sea salt aerosol and we propose that these values should be used as a baseline reference in future studies.”
Comment: Although the authors explicitly state that they are dealing with ”inorganic sea salt particles”, as reflected in the title, they mention ”sea spray particles” and ”inorganic sea spray particles” several times in the text. Sea spray particles are generally considered those that contain organics in addition to sea salt. Thus perhaps restricting discussion only to ”sea salt particles” or ”inorganic sea salt particles” might be advisable to remove any possibility of confusion.
Reply: We agree and have changed “sea spray” to ”sea salt” where appropriate. We have kept the wording ” sea spray” (which includes the inorganic as well as the organic part) in the first part of the introduction until we specifically mention that this work focuses on the inorganic part of the sea spray particles which is known as sea salt. We have also modified the second sentence of the introduction to make this differentiation more clear:
“These particles consist of a mixture of inorganic salts, here termed sea salt particles, and organic compounds and are generated at the ocean surface (we term the mixture of inorganic and organic compounds sea spray aerosol)”.
Comment: The term “hygroscopicity” is used throughout the document, but in different ways. The colloquial meaning (such as used on lines 25, 27, 30, 32, and elsewhere) refers to the ability of a particle to uptake water substance. However, throughout the manuscript this term was used in a more quantitative manner to refer to a numerical value. Perhaps it would be preferable to use the term in only one sense or the other.
Reply: We agree and have unified the wording throughout the manuscript using “hygroscopicity” for the effect of water uptake and the “hygroscopic growth” or “hygroscopic growth factor” when referring to the actual numerical values. We continue to call the κ-value the hygroscopicity parameter κ.
Comment: On line 67 and thereafter, the authors stated that NaCl transitioned from almost cubical to nearly spherical, with smaller particles having more rounded edges. While this explanation might be consistent with a dynamical shape factor more near unity, it seems to be merely an assumption, and not a conclusion, as it was stated.
Reply: Rather than an assumption or conclusion, this statement was intended to summarise the microscopy-based conclusions of Zelenyuk et al. (2006), as cited in the previous sentence. For clarification, we have changed lines 69–70 to:
”This observation is in accordance with the electron micrographs of Zelenyuk *et al.*[13], who observed rounder edges on smaller nebulized-NaCl particles, which corresponds to smaller shape factors.”
Comment: Artificial sea salt is well known to contain additives to reduce clumping, and sometimes organic substances also. Was the mixture baked before use to remove organics?
Reply: The sea salt was not baked since this process irreversibly changed the morphology of the sea salt crystals and likely their chemical composition. However, in Salter et al. (2016) we used both X-ray photoelectron spectroscopy (XPS) and vibrational sum frequency spectroscopy (VSFS) to test whether surface active organic matter was present in artificial sea salt. Both surface-sensitive methods did not observe any surface active organics. We also contacted the manufacturer, who stated that there is no anti-caking agent added to the salt (Quote from email of Merck’s Technical Service: ”Our product manager has advised that the anti caking agent would be calcium phosphate and that is not present in our product as there is no phosphate present. In otherwords, the anti caking agent is not used in our S9883 product.”). Therefore, we believe that the artificial sea salt is free of organic substances. For clarification we have added to the method section:
”In Salter *et al.*[24] we used both X-ray photoelectron spectroscopy (XPS) and vibrational sum frequency spectroscopy (VSFS) to test whether surface active organic matter was present in artificial sea salt. Both surface-sensitive methods did not observe any surface active organics. In addition, the manufacturer states that no anti-caking organics are used in the production of the salt mixture. Therefore, organic substances would only have been present in similarly low concentrations (below detection limit) across all of our measurements which means any effect on the hygroscopic growth will have been negligible.”
Comment: How was the dynamic shape factor for a perfect cube as a function of mobility diameter determined (Figure 1)? This value should asymptote to 1.08 for large Dmob, and should attain the value 1.24 for very small Dmob. Perhaps these could be shown on the figure.
Reply: The plotted function is the Dahneke adjusted-sphere interpolation of the asymptotic values 1.08 and 1.24. We realised that this was not stated in the original manuscript and thank the reviewer for pointing it out. To clarify we changed lines 63–64 to:
“The curves corresponding to perfect spheres (unity) and perfect cubes (using the Dahneke adjusted-

sphere interpolation[13] as detailed in Biskos et al.[14]) are also shown."

In response to the reviewer's suggestion, we have also extended the plotted function to cover the full range of particle sizes plotted in Fig. 1 (main manuscript).

Comment: Why is the ADDEM result expected to be higher (line 117)? There was no previous discussion of this model before this statement.

Reply: ADDEM for NaCl is expected to be higher since our measurements of inorganic sea spray are lower than the measurements for NaCl. However, we agree that the 'as expected' is slightly misleading at this point since now modelled values are compared to the measurements. We therefore have removed these two words ('as expected'). In addition, we have added a sentence to guide the reader to the method section, where all the thermodynamical models are described:

"Further details on the different models used can be found in the method section."

2 Reviewer #2 (Remarks to the Author)

Comment: This laboratory study investigates and revises the hygroscopicity of inorganic sea salt aerosol, an important parameter used in global modeling of aerosol effects on climate. It is detailed and well written and should be accepted to Nature Communications. The majority of my comments are minor, but I would like the authors to consider discussing the implications of their work in the context of internally mixed particles. Their laboratory work focuses on the inorganic component of sea salt particles, but how important is it also to consider the organic component? Would considering this component even further reduce hygroscopic growth and be important for climate models to consider? I would suggest to consider this in the discussion and impacts section.

Reply: In first order approximation the total water associated with sea spray particles is the sum of the water associated with the inorganic components and that associated with the organic components, be it an internal or an external mixture of the two. Although modelling studies have suggested that the presence of organics in sea spray acts to reduce its hygroscopicity (Ming and Russell, 2001), recent measurements suggest that organic substances do not significantly reduce the hygroscopicity of nascent sea spray aerosol (Collins et al., 2016; Nguyen et al., 2017). As such, to understand the hygroscopicity of sea spray aerosol it is critical that we first understand the inorganic fraction which dominates the water uptake. Further, our work is centred around the inorganic component of the sea salt since it is generally modelled separately in GCM's. To clarify these issues we have added the following sentence:

"In the ambient atmosphere, the contribution of organic substances may further decrease the hygroscopicity of the ambient sea spray particles[37]."

In addition we have added to the last paragraph of the impact section (where we discuss the model results): "Although models suggest that the presence of organics in sea spray acts to reduce its hygroscopicity[37] recent measurement results suggest that organic substances do not significantly reduce the hygroscopicity of nascent sea spray aerosol[38,39]. As such, to understand the hygroscopicity of sea spray aerosol it is critical that we first understand the inorganic fraction which dominates the water uptake."

Comment: Below I give only minor comments:

1. Abstract: "The particle generation method is an important factor for hygroscopicity measurements since it determines the particle's shape and chemical composition. We report, for the first time, size-dependent hygroscopic growth for particles smaller than 150 nm in diameter. This observation is independent of the particle generation method, and likely caused by size-dependent changes in particle solubility or surface composition in the submicrometer particle range."

I found these sentences a bit confusing to read the first time since you first say particle generation method is important then seem to contradict this in the third sentence above. Consider rewording somehow to make your meaning more clear.

Reply: We agree and have changed the second part to:

"The size-dependent increase in hygroscopic growth with decreasing particle size is independent of the particle generation method, and is likely caused by size-dependent changes in particle solubility or surface composition in the submicrometer particle range."

Comment: 2. Avoid using "state-of-the-art" buzzword - I'm sure all models consider themselves so!

Reply: We agree and have removed it throughout the manuscript.

Comment: 3. Fig 2. Consider plotting full theoretical curve for NaCl (hydration and dehydration)

Reply: We agree and have plotted the full theoretical curve (in panel b).

Comment: 4. Line 117: Suggest a short sentence describing the models, or point to section where this occurs

Reply: We have added the following sentence to guide the reader to the relevant section in the methods, where all the models are described in detail. "Further details on the different models used can be found in the method section."

Comment: 5. Fig 4 and S4: Please add global mean on 2D plots; also, the notation of title is confusing as at first I thought Fnet was 1.5 W/m2 (not that K = 1.5).
Reply: We agree and have added the global mean values to the caption of the Figures 4, S4 and S5. We also changed the notation in Fig. S4 for clarity.
Comment: 6. In your discussion you imply inorganic part is most important (over organic) for reduced hygroscopicity, but you did not test this so how do you know?
Reply: Within this work, we focus on the inorganic fraction of sea spray aerosol for the reasons described in our response to this reviewers major comment (see above). In the discussion, we are not comparing the importance of the inorganic and organic composition for hygroscopic growth. Rather, through the use of three independent methods, and the use of artificial sea salt with minimal organic contamination, we have probed the influence of the inorganic composition on the hygroscopicity of sea spray as a first step to understanding the hygroscopicity of sea spray aerosol. If we cannot understand the hygroscopicity of the sea salt itself, understanding the complex mixture of inorganics and organics that is sea spray will be impossible. As such, following our detailed characterisation of the inorganic sea component of sea spray aerosol, future studies will focus on characterising the effect of organic substances on the hygroscopicity of sea salt aerosol.
Comment: 7. Very interesting that the global model accidentally was using the "right" K. I wonder if other models did similarly? Maybe look at AeroCom results?
Reply: This is indeed interesting. However, there are many different parametrisations of aerosol hygroscopicity used by GCM's and there is, to our knowledge, no conclusive summary for AeroCom models. Despite this, we are aware that there are sevaral models appart from ECHAM that use directly or indirectly the values of Tang et al. (1997) to parameterize the hygroscopicity of inorganic sea salt particles (e.g. NASA's Geophysical Fluid Dynamics Laboratory Global Atmospheric Model (GFDL) GCM; Paul Ginoux, personal communication, October 2016). All models that use the OPAC database by Hess et al. (1998) for their aerosol scheme (e.g., NASA's Goddard Chemistry Aerosol Radiation and Transport (GOCART) model) are affected since their sea salt component is assumed to be NaCl (see Tab. 4 in Zieger et al., 2013). We are planning to look into the hygrosopicity representations in climate models in a more comprehensive way in a future study.

3 Reviewer #3 (Remarks to the Author)

General comments: This study is primarily based on measurements of the hygroscopicity of both submicrometer (humidified tandem differential mobility analyzer, HTDMA) and super-micrometer (electrodynamic balance, EDB) inorganic sea salt aerosol particles generated in laboratory from artificial inorganic sea salt solutions assumed as sea water by the authors. The study seems technically sound, but rather incremental than novel. The measurement methods used are well known to the community, but have been executed meticulously. The major claim by the authors in this study is that "the measured reduction in the inorganic sea salt aerosol hygroscopicity, κs, from 1.5 (NaCl, considered as model sea salt) to 1.1 (NaCl with other salts like MgCl2 and CaCl2) manifested in a decrease of up to ~15% in aerosol optical depth (AOD), while indirect or cloud related effects were practically insensitive to this change". The conclusions are limited by assumptions that sea spray aerosol (SSA) particles are composed of the inorganic sea water components only. On the other hand, several previous reports have proven beyond doubt that fresh sea-spray particles either collected from remote marine locations or generated in the presence of sea surface planktons in the laboratory or ocean-atmosphere facilities are dominated by organics at the submicron level [1, 2, 3, 4, 5]. Furthermore, the inorganic components react within very short time leading to depletion of chloride and formation of nitrates and/or nss or non-sea-salt sulfates, etc.[5, 6, 7, 8]. It is not clear how the results from this study can be used or extended towards the realistic parametrization (hygroscopicity and optical properties) of the more chemically complex sea-spray particles on a global scale and hence raises doubts about its impact on the wider atmospheric research community. The results, however, may provide some insight to researchers, specialized in the field, for future studies on the hygroscopicity of the inorganic components in real, ambient, marine aerosol species. I have my doubts whether the current version of the manuscript is publishable in nature-communications. I would suggest more specialized journals in this field. The authors need to address following major concerns in order to create greater impact.

Major Comments:
Comment: 1. Continuing from the general comments above, the authors need to make it clear how the results from this study can be used or extended towards the parametrization (hygroscopicity and optical properties) of the real and more chemically complex sea-spray particles, which they claim are not easy to validate for a number of reasons as given in Pg. 2, lines 39-45.
Reply: We agree with the reviewer that sea spray aerosol is a complex mixture of inorganic salts and organic compounds. It is this complexity that makes understanding the hygroscopic growth of sea spray aerosol particles so difficult. Our study, which focuses solely on the inorganic part of the sea spray aerosol, is important for several reasons:

1. Measurements of the hygroscopic growth of ambient marine aerosol and nascent sea spray aerosol generated in laboratory experiments are uniformly compared to NaCl as a reference substance. Our study highlights that this approach is invalid and that referencing to artificial sea salt that contains the relevant hydrates is a more sound approach. Comparing to NaCl will likely overstate the importance of the organic components in sea spray aerosol in reducing the hygroscopic growth. This is interesting in the light of recent measurements that suggest marine biota has a limited effects on the CCN activity of nascent sea spray aerosols (Collins et al., 2016).

2. Rightly or wrongly, all state-of-the-art large scale models parametrise sea spray aerosol as two discreet components, the sea salt fraction and the organic fraction. Therefore, if the inorganic sea salt component does not have the correct hygroscopic growth, the output affected by these values will be inaccurate.

In summary, in order for the field to understand the hygroscopicity of sea spray aerosol (organics and inorganics), we must have a full comprehension of the hygroscopicity of inorganic sea salt. This study provides essential reference data for future climate models as well as detailed studies on the impact of organics on sea spray hygroscopicity.

Comment: 2. How do the authors assume that the inorganic composition of the sea salt aerosol particles is primarily responsible for the suppression of the hygroscopic growth below NaCl rather than organic contamination (Pg. 4, lines 190-192)? Did the authors find comparable values of hygroscopicity parameter κs in literature (e.g. for Modini et al. in Table S1)? This and other such instances involving hygroscopicity of particles generated from natural sea-water should be highlighted and quantitatively stated in the main text.

Reply: In Salter et al. (2016), we probed the pure inorganic sea salt mixture for organic contamination, which could not be detected by two different methods (vibrational sum frequency spectroscopy (VSFS) and X-ray photoelectron spectroscopy (XPS)). Both surface-sensitive methods did not observe any surface active organics. Therefore, organic substances would only have been present in similarly low concentrations (below detection limit) across all of our measurements which means any effect on the hygroscopic growth will have been negligible. Importantly, we used three independent methods to access the hygroscopicity of inorganic sea salt particles and all three techniques showed a consistent lower hygroscopic growth compared to NaCl, which strengthens our conclusions. In addition, the size-resolved measurements of the HTDMA show a size dependence of g_e(RH) which has not been reported before.

Another important part of our work is that we directly measured the shape of the particles as well as their (dry) density, which, to our knowledge, none of the previous studies have done. The shape factor, which directly influences the magnitude of the hygroscopic growth factor, had to be assumed or was not considered at all in previous studies. A comparison to literature values of the hygroscopicity of pure inorganic sea salt particles is provided in Table S1 and highlights that, although many studies present values, these studies lack important auxiliary measurements (like the shape factor) or fail to report dry reference RH. The hygroscopic growth factor g_e(RH) is a physically-based property, while κ is only a semi-empirical parameter which can be calculated from it. We therefore believe that we should compare our findings to the directly measured hygroscopic growth factors g_e(RH) found in the literature as presented in Table S1.

Comment: 3. The authors state that "if the surface composition is driving the water uptake at small sizes, only minute amounts of surface-tension-reducing substances are needed to result in a notable effect" (Pg.3, line 137). Which inorganic components (artificial sea water), with reduced surface tension, do the authors think can partition to the surface of the smallest particles and cause the observed increase in water uptake beyond thermodynamic predictions? How would organic contaminants, especially surface active organics and/or water insoluble organics, affect these small particles? Some clear arguments here can benefit the readers more than just cited literature.

Reply: In Salter et al. (2016) we have reported that calcium is enriched in the submicron particle size range. The enrichment was observed with the same (size-dependent) magnitude independent of the type of seawater used (artificial seawater, as used in this manuscript, and one ambient ocean water sample) and exhibited similar behaviour, as a function of particle size, to the particle surface-to-volume ratio. Therefore, we proposed that non or only minor amounts of organic contaminants are needed to enrich calcium at the droplet surface. We repeat this argument in the revised manuscript by adding the following sentences:

"Salter et al.[21] observed Ca^{2+} in nascent sea spray aerosols using both the same artificial sea salt solution used in this study and one ambient seawater sample. Both seawater samples showed the same size-dependence and magnitude in Ca^{2+}-enrichment, independent of the amount of organic carbon in the seawater. As such Salter et al.[21] concluded that the enrichment in Ca^{2+} may not require the presence of organics. Taken further, these observations may, at least partially, explain our observations of increasing hygroscopicity with decreasing particle size in submicrometer inorganic sea salt particles."

We have also estimated the surface tension reduction needed to explain the increase in hygroscopicity with decreasing particle size: for particles otherwise resembling sodium chloride, the change of hygroscopicity between 150 and 50 nm would require a surface tension reduction in the order of several tens of mNm^{-1}.

This is obviously a rather substantial reduction, but is, in principle, possible if e.g. small amounts of highly surface active contaminants were present on the surface. It should be stressed, however, that in our previous studies on the mixtures, no evidence of such surfactants were observed.

Comment: 4. The measured growth factors ge(RH =90%) for pure NaCl particles have been plotted in Figure 3 and listed in Table S1. It would be better if authors include the hygroscopicity parameter κs values for NaCl calculated from these experimentally measured values in Table 1 along with the theoretical values. That should strengthen the authors' claim that the particle generation methods are not responsible for the measured reduction in hygroscopicity of sea-salt particles compared to NaCl and showcase better reproducibility of the measurement conditions.

Reply: We agree and have added the measured growth factors of NaCl (HTDMA and EDB) and the calculated κ_s-values to Table 1. In addition, we clarified (at various points throughout the manuscript) that κ_s was calculated at RH=90%. In Table S1, we would like to show only the reported and physically more relevant hygroscopic growth factors, while we show (and discuss) the κ_s-values in Table 1 in the main manuscript to make our point that the change in chemical composition is likely responsible the difference in hygroscopic growth with varying dry particle size and particle production method (jet vs. nebulizer).

Comment: 5. (Pg.5 lines 221-225) How do the authors ensure that particles are completely dry (5-8% in HTDMA and < 3% in EDB as listed in Table S1) given that sea-salt aerosols and MgCl2 particles reportedly exhibited presence of residual water at RHs < 2% [9, 10, 11]? Were the hydration-dehydration cycles repeated? Is there a possibility that the laboratory generated droplets when passed through diffusion dryer may exhibit kinetic artefacts (restructuring) due to fast drying [12]. What could be the effect on shape factors when particles are dried through the diffusion dryer compared to slow equilibrated dehydration in the experimental loop?

Reply: This is an important comment. Even at very dry RH conditions (as in our experiment), particles will always contain remaining water. As pointed out in more detail by reviewer 1, this will affect the dry density and the retrieval of the hygroscopic growth factors. We have therefore re-confirmed that the dry sea salt mixture contained water (using FTIR) and have re-measured the dry density using a helium-pycnometer. We emphasise that the remaining water is an important component of the inorganic sea salt since it will be present in sea spray aerosol in the atmosphere. Many ambient aersosol measurements (e.g., HTDMA, VT-DMA or filter measurements) are affected by these hydrates, as were the measurements by Tang et al. (1997), who likely corrected for the remaining water in their main figure. We would also like to emphasise that although the corrected value presented by Tang et al. (1997) is not atmospherically relevant, it has been used to represent the hygroscopicity of sea salt in atmospheric models. We have extended the discussion on the remaining water (hydrates) in the revised manuscript due to it's importance.

Concerning the second part of the question: In HTDMA measurements we didn't vary the drying time of the particles, but the drying took place relatively fast (in approx. 2 seconds). The EDB cycle was performed once on the same particle due to its long measurement time (approx. 56 hours for a full cycle). Concerning the artifacts due to drying: We have kept the flow regime and residence times in the diffusion driers as identical as possible for the HTDMA and shape factor measurements to avoid effects (which we also properly mention and cite in the original manuscript, see last sentences in the section 'Sea salt particle generation methods'). The very slow dehydration cycle of the EDB (approx. 28 hours) shows the same curvature of g(RH) compared to the fast HTDMA measurements (see Fig. S3). That the three independent measurement techniques used agree so well (HTDMA, EDB and water activity) suggests that kinetic artifacts are negligible.

Comment: 6. Authors claim that "finally, the climate impact of our results are illustrated using a state-of-the-art large-scale model" (Pg.2, Line 58). Plots of the AOD (Figure 4 and S5) estimated by running ECHAM6-HAM2 global climate model (GCM) considering reduced hygroscopicity parameter κs = 1.1 for sea salt particles (instead of 1.5) does show a significant reduction. How does the GCM obtained AODs compare with the real situation (at least some measured regional AODs, say in the Southern Ocean)? Authors should tone down their claims as the several studies on marine aerosol composition (cited in general comments) conducted post Tang et al [13] clearly indicates that sea-spray aerosols are evidently more complex and NaCl or just pure sea-salt (NaCl with other salts of Mg, Ca, etc.) may no longer be suitable representatives. A discussion may be included on whether GCMs need to be modified in order to accommodate different classes of sea spray aerosols with low uncertainty in hygroscopicity for each class. How does the constrained hygroscopicity parameter κs = 1.1 represent particles with organic contaminants which might have much lower hygroscopicity? How realistic is this applied constraint? If authors think that pure sea salt particles have an important/realistic role in the parametrization, relevant enough to the marine atmosphere, they should mention the abundance of such particles vis-a-vis other types containing organic and/or reacted species (as mentioned in general comments) citing literature.

Reply: The AOD modelled by ECHAM-HAM has been shown to compare well with observations (see e.g. Fig. 1 in Schutgens et al., 2016, who uses the same model version as in our work). As pointed out by reviewer 1, the choice of the sea salt source parametrisation has a strong influence on the AOD. We have tested an-

other source parametrisation by Long et al. (2011) in ECHAM (see Fig. 2, which is also added to the revised supplement). Both parameterizations are distinct different in size-dependent source strength (see Fig. 5a in Salter et al., 2015). There are clear differences in the AOD as a function of latitude between the model runs using the different sea salt source functions and these differences are especially pronounced in the southern hemisphere where the influence of sea spray is strongest. However, despite these differences, the relative changes when reducing κ_s from 1.5 to 1.1 are essentially the same (a decrease of up to $15-17\,\%$ in the southern hemisphere) whichever sea salt source function is used. As such our main point is unchanged: the hygroscopic growth of the inorganic sea spray component is one important determining factor of the AOD and the direct radiative forcing among many others (e.g. source function or the various removal parametrisation).

Our study is solely focused around the inorganic part of sea spray aerosol. The reviewer is correct that there are many post-Tang studies that show the complexity of sea spray aerosol with ambient or lab-based measurements. However, all these studies include the organic fraction in their analysis and interpretation which makes direct comparison to our work impossible. We show that, in terms of hygroscopicity, it is not appropriate to assume that NaCl is representative for the inorganic fraction of sea spray aerosol. Most models represent nascent sea spray aerosol as two separate components, one for the inorganic and one for the organic mass fraction. Although, this is clearly an oversimplification, while models are constructed in this fashion there is clearly a need to understand the behaviour of the inorganic fraction. This was the aim of our study with the result that our proposed κ_s is only representative for the inorganic part of the sea spray aerosol.

With regards the comment on organic compounds, although models routinely predict reduction in the hygroscopicity of sea salt particles if organics are present, recent measurements of the hygroscopicity of nascent sea spray aerosol (containing organics) suggest very little impact (Collins et al., 2016; Nguyen et al., 2017). This suggests that the hygroscopicity of nascent sea spray aerosol is driven by the inorganic compounds. Therefore, to correctly predict the hygroscopicity of sea spray aerosol understanding the dominating inorganic component is critical.

Comment: Specific Comments: 1) (Pg.1, line 30). A sentence or two about the implications on heterogeneous chemistry due to the presence of condensed water over a wider RH range in sea salt aerosols compared to NaCl, citing literature [14, 15], may be added in the "discussion and impact" section.

Reply: Water uptake by sea salt particles at intermediate RH-values has been shown in many previous publications and is well known to the scientific community. Therefore, we do not see a need to discuss this particular aspect again. Reference 14 is cited in the result section where the deliquescence behaviour is discussed.

Comment: 2) (Pg.2, line 46) Please simplify the sentence for better clarity.

Reply: We agree and have made three separate sentences out of the previous one: "The variability in terms of water uptake of aerosol particles among major large-scale models is still large[12]. One reason for this disagreement is differences in the assumptions made by each hygroscopicity parametrisation implemented. This highlights the need to better constrain hygroscopicity parametrisations."

Comment: 3) (Pg. 2, lines 77-79 and Pg.3, line 93-94) A mutual deliquescence of the MgCl2-dominant eutonic component in NaCl/MgCl2 mixed particles at RH = 15-16% was reported in recent literature [14] which can be cited here. The final DRH value due to the major salt, NaCl should be dependent on the mole fraction of NaCl in the salt mixture and hence should be slightly lower than that of pure NaCl (75.3% at 25°C).

Reply: We agree and have modified this part. "These partially dissolved states, known as eutectics or mutual deliquescence[15], were also observed by Tang *et al.*[10] and recently by Gupta *et al.*[16]. NaCl exhibits its main deliquescence RH (DRH) at RH = $74.3 \pm 1.5\,\%$ (complete dissolution of NaCl), while the main DRH of the inorganic sea salt mixture is slightly shifted to lower RH (~73.5 %)."

Comment: 4) (Pg.3, lines99-100) Check the closing parenthesis "(although 3.5% lower) than the...)".

Reply: We have corrected the misplaced parenthesis.

Comment: 5) (Pg.5, shape factor measurements) What is the percentage contribution of shape factor (χ) uncertainty in the ge and κ_s calculations? In Figure 1, the uncertainty in χ_t for nebulized smallest particles seems larger than that of large-mass particles. What could be the reason?

Reply: The variability over repeated dynamic shape factor measurements was much larger than the statistical uncertainty of each measurement, so the larger error bars indicate greater experimental variability, not greater uncertainty. The error bars in Figure 1 depict this variability, using the standard deviation of repeated measurements and show that it was $< 3\,\%$ for all three experiments.

We do not know why the smallest nebulized sea salt aerosols exhibit a larger variability in their measured values. However, we can offer the conjecture that variation in particle shape for more-highly-aspherical particles (higher χ) results in a variation in χ that is measurable, whereas for near-spherical particles with $\chi \approx 1$, variations in particle shape lead to only minor variations in χ relative to uncertainty.

The error propagation was recalculated by combining, in quadrature, the instrumental uncertainty of HTDMA with the uncertainty of shape factor correction (maximum 2 %, corresponding to the 3 % s.d. of re-

peated shape factor measurements). We have added this information to the manuscript (method section) and updated the figures accordingly (the error bars have slightly increased). "An overall measurement uncertainty of 5.5 % (upper limit for all three HTDMA experiments) was estimated by combining, in quadrature, the instrumental uncertainty of the HTDMA (max. 4.7 %) with the uncertainty of the measured shape-factor corrections (max. 2 %, from the standard deviation of repeated measurements)."

References (reviewer 3)

1. Facchini MC, et al. Primary submicron marine aerosol dominated by insoluble organic colloids and aggregates. Geophys Res Lett 35, L17814 (2008).

2. Quinn PK, et al. Contribution of sea surface carbon pool to organic matter enrichment in sea spray aerosol. Nature Geosci 7, 228-232 (2014).

3. Quinn PK, Collins DB, Grassian VH, Prather KA, Bates TS. Chemistry and Related Properties of Freshly Emitted Sea Spray Aerosol. Chem Rev, (2015).

4. Ault AP, et al. Size-Dependent Changes in Sea Spray Aerosol Composition and Properties with Different Seawater Conditions. Environ Sci Technol 47, 5603-5612 (2013).

5. Prather KA, et al. Bringing the ocean into the laboratory to probe the chemical complexity of sea spray aerosol. Proc Natl Acad Sci 110, 7550-7555 (2013).

6. Ault AP, et al. Heterogeneous Reactivity of Nitric Acid with Nascent Sea Spray Aerosol: Large Differences Observed between and within Individual Particles. J Phys Chem Lett 5, 2493-2500 (2014).

7. Ault AP, et al. Inside versus Outside: Ion Redistribution in Nitric Acid Reacted Sea Spray Aerosol Particles as Determined by Single Particle Analysis. J Am Chem Soc 135, 14528-14531 (2013).

8. Laskin A, et al. Reactions at Interfaces As a Source of Sulfate Formation in Sea-Salt Particles. Science 301, 340-344 (2003).

9. Tang IN, Tridico AC, Fung KH. Thermodynamic and optical properties of sea salt aerosols. J Geophys Res – Atmos 102, 23269-23275 (1997).

10. Cziczo DJ, Nowak JB, Hu JH, Abbatt JPD. Infrared spectroscopy of model tropospheric aerosols as a function of relative humidity: Observation of deliquescence and crystallization. J Geophys Res – Atmos 102, 18843-18850 (1997).

11. Cziczo DJ, Abbatt JPD. Infrared Observations of the Response of NaCl, MgCl2, NH4HSO4, and NH4NO3 Aerosols to Changes in Relative Humidity from 298 to 238 K. J Phys Chem A 104, 2038-2047 (2000).

12. Mikhailov E, Vlasenko S, Martin ST, Koop T, Pöschl U. Amorphous and crystalline aerosol particles interacting with water vapor: conceptual framework and experimental evidence for restructuring, phase transitions and kinetic limitations. Atmos Chem Phys 9, 9491-9522 (2009).

13. Tang IN. Thermodynamic and optical properties of mixed-salt aerosols of atmospheric importance. J Geophys Res – Atmos 102, 1883-1893 (1997).

14. Gupta D, Eom HJ, Cho HR, Ro CU. Hygroscopic behavior of NaCl-MgCl2 mixture particles as nascent sea-spray aerosol surrogates and observation of efflorescence during humidification. Atmos Chem Phys 15, 11273-11290 (2015).

15. Gupta D, Kim H, Park G, Li X, Eom HJ, Ro CU. Hygroscopic properties of NaCl and NaNO3 mixture particles as reacted inorganic sea-salt aerosol surrogates. Atmos Chem Phys 15, 3379-3393 (2015).

4 Further changes

- We removed panel b in Fig. S3 since it is already shown in the manuscript.
- We have modified the manuscript to meet the editorial requirements of Nature Communications. The main changes are:
  - New colour schemes to avoid red and green to be at the same time in one figure.
  - We have re-written the abstract to reach the limit of 150 words as required by Nature Communications.
  - We changed the last paragraph of the introduction to meet the requirements of the Nature Communications (Checklist: "The last paragraph contains a brief summary of both the results and the conclusions (written in present tense)").
  - We added titles to the figures and tables.
  - We removed the notes in the supplementary material since all required information is given in the figure captions.

References

Collins D.B., Bertram T.H., Sultana C.M., Lee C., Axson J.L., and Prather K.A., Phytoplankton blooms weakly influence the cloud forming ability of sea spray aerosol, *Geophys. Res. Lett.*, 43(18), 9975–9983, 2016.

Cziczo D., Nowak J., Hu J., and Abbatt J., Infrared spectroscopy of model tropospheric aerosols as a function of relative humidity: Observation of deliquescence and crystallization, *J. Geophys. Res.*, 102(D15), 18843–18850, 1997.

Gong S., A parameterization of sea-salt aerosol source function for sub-and super-micron particles, *Global Biogeochem. Cycles*, 17(4), 1097, doi:10.1029/2003GB002079, 2003.

Hess M., Koepke P., and Schult I., Optical properties of aerosols and clouds: The software package OPAC, *Bull. Amer. Meteor. Soc.*, 79(5), 831–844, 1998.

Long M.S., Keene W.C., Kieber D.J., Erickson D.J., and Maring H., A sea-state based source function for size- and composition-resolved marine aerosol production, *Atmos. Chem. Phys.*, 11(3), 1203–1216, doi:10.5194/acp-11-1203-2011, 2011.

Ming Y. and Russell L., Predicted hygroscopic growth of sea salt aerosol, *J. Geophys. Res.*, 106, 28259–28274, doi:10.1029/2001JD000454, 2001.

Modini R.L., Harris B., and Ristovski Z.D., The organic fraction of bubble-generated, accumulation mode Sea Spray Aerosol (SSA), *Atmos. Chem. Phys.*, 10(6), 2867–2877, doi:10.5194/acp-10-2867-2010, 2010.

Nguyen Q.T., K.H.K., Kling K.I., Boesen T., and Bilde M., Impact of fatty acid coating on the CCN activity of sea salt particles, *Tellus B (in press)*, 2017.

Rasmussen B.B., Nguyen Q.T., Kristensen K., Nielsen L.S., and Bilde M., What controls volatility of sea spray aerosol? Results from laboratory studies using artificial and real seawater samples, *J. Aerosol Sci.*, pages –, doi:http://dx.doi.org/10.1016/j.jaerosci.2017.02.002, 2017.

Salter M.E., Hamacher-Barth E., Leck C., Werner J., Johnson C.M., Riipinen I., Nilsson E.D., and Zieger P., Calcium enrichment in sea spray aerosol particles, *Geophys. Res. Lett.*, 43(15), 8277–8285, doi:10.1002/2016GL070275, 2016GL070275, 2016.

Salter M.E., Zieger P., Acosta Navarro J.C., Grythe H., Kirkevåg A., Rosati B., Riipinen I., and Nilsson E.D., An empirically derived inorganic sea spray source function incorporating sea surface temperature, *Atmos. Chem. Phys.*, 15(19), 11047–11066, doi:10.5194/acp-15-11047-2015, 2015.

Schutgens N.A.J., Partridge D.G., and Stier P., The importance of temporal collocation for the evaluation of aerosol models with observations, *Atmos. Chem. Phys.*, 16(2), 1065–1079, doi:10.5194/acp-16-1065-2016, 2016.

Tang I.N., Tridico A., and Fung K., Thermodynamic and optical properties of sea salt aerosols, *J. Geophys. Res.*, 102(D19), 23269–23275, 1997.

Zelenyuk A., Cai Y., and Imre D., From agglomerates of spheres to irregularly shaped particles: Determination of dynamic shape factors from measurements of mobility and vacuum aerodynamic diameters, *Aerosol Sci. Technol.*, 40(3), 197–217, 2006.

Zieger P., Fierz-Schmidhauser R., Weingartner E., and Baltensperger U., Effects of relative humidity on aerosol light scattering: results from different European sites, *Atmos. Chem. Phys.*, 13(21), 10609–10631, doi:10.5194/acp-13-10609-2013, 2013.

REVIEWERS' COMMENTS:

Reviewer #1 (Remarks to the Author):

Review of NCOMMS-16-24262A, a revision of "Revising the hygroscopicity of inorganic sea salt particles," by P. Zieger et al., submitted to Nature Communications.

The authors have done an admirable job of incorporating numerous comments from all three reviewers, and they have an improved and very good manuscript. The work should certainly be published, but I echo the sentiments of Reviewer 3 that although the work is "technically sound," it is "rather incremental than novel," and as such "would suggest more specialized journals." I don't think that Nature Communications is the appropriate venue for this work – it is just too specialized. I were to be asked to review this manuscript for Journal of Geophysical Research or Atmospheric Chemistry and Physics, I would unhesitatingly recommend accept immediately.

I have a few comments to propose to the authors to further strengthen this manuscript.

On p. 7 of the revised manuscript, it is stated "to convert mass growth to size growth, we proceeded as described in Zardini et al." However, this requires the reader to look up this article, and the authors could have explained what they did very simply by stating "radial hygroscopic growth was determined from mass hygroscopic growth using the assumption that the volumes of water and solute are additive," which is what Zardini assumed. That said, the authors can do better: as they know the density as a function of relative humidity (from Tang), they can actually calculate explicitly the radial growth without making the assumption that the volumes are additive. This approach would eliminate the uncertainty introduced by the assumption of volume additivity. It might not be large, but the point of the entire manuscript is that details of this nature are important. The relative importance of this assumption can be found by comparing the density of seawater with the sum (1-x)/rho_water + x/rho_seasalt (x is the solute mass fraction), which is what the density would be if volume additivity held.

The authors did the extra step, which in and of itself is an important contribution to the literature, of measuring the density of sea salt, from which they obtained the value ~2.02 g/cm^3. From this value, they argued that the lower bound suggested by this reviewer in his comments on the first version of the manuscript was too low. Might the measured value provide some insight into which substance(s) contains hydrates? Is it $MgCl_2$, or $CaCl_2$, or Na_2SO_4? This would be an interesting addition to the manuscript, and I would think that some discussion of this would be value added.

A dozen lines or so before "Results," it might be advisable to mention that "hygroscopic growth" refers to diameter. This is the first mention of this in the manuscript, and although specialists in the field know that the term refers to radial growth (i.e., one dimension), a general audience won't know if this refers to mass or diameter.

The responses to the reviewers summarizes the work in four bullets, with two additional bullets describing what is new. This is an excellent summary of the work, which I would suggest that they incorporate into the manuscript.

Finally, it is not clear in the supplemental figure 2 why the Kelvin corrections have different coefficients (-5.89 vs -4.71) in the term 1/Ddry. In the text on p. 8 I could see that the authors modified the value 6 to 4.71, but I wasn't explained where the 5.89 came from.

Reviewer #2 (Remarks to the Author):

I am satisfied with the authors' reviewer responses and recommend publication.

Reviewer #3 (Remarks to the Author):

Review report on the revised manuscript NCOMMS-16-24262A entitled "Revising the hygroscopicity of inorganic sea salt particles".

General comments
In general, the manuscript has been thoroughly revised and the authors have responded to the reviewers' comments satisfactorily. The results provide valuable insight on a very specific but important aspect i.e. the hygroscopicity of inorganic sea-salt component in marine aerosol species. The authors claim to have established that inorganic sea-salt show 8-15 % lower hygroscopic growth than pure NaCl, which has so far been considered for the parameterization of sea-salt hygroscopicity in circulation models. The work has been meticulously executed. Supporting measurements (FTIR and pycnometer) have been done to address the important question regarding dry-particle mass/density due to the possible presence of hydrates at low RH, in the revised version. In the body of the original manuscript the reference to sea-spray aerosols instead of inorganic sea-salt particles was confusing, as was rightly pointed out by Reviewer 1. It has been addressed in the revision. The authors have also done well to clarify their claims by saying that this work is a small but important step towards understanding the hygroscopic and optical properties of the more chemically complex sea-spray aerosols, which are invariably internally mixed with organics. The quality of the manuscript and clarity of purpose has improved after the revision and I recommend that the work should be published. On the other hand, I have my doubts whether it meets the novelty standards to be published in "nature-communications".
Suggested rephrasing of sentences in Pg. 5, paragraph 4: "Inorganic sea-salt "component contains" water even at very low RH as an integral part of the crystalline structure. "The dominant hydrates may possibly be" MgCl2·6H2O and CaCl2·10H2O".
Some further minor points to think about here and in reference to the modifications done in the revised manuscript i.e. regarding sea-salt particle drying in response to the questions pointed out mainly by reviewer 1 and 3 (major comments #5):
How, do the authors know for sure that only the thermodynamically stable hydrates (MgCl2·6H2O and CaCl2·10H2O) are formed in dispersed particles? Are the FTIR spectra enough to conclude on these particular hydrate structures? Were the FTIR and density (pycnometer) measurements done on bulk deposited particles generated by both nebulization and from the chamber? Cziczo et al used dispersed aerosols in a flow tube for FTIR measurements and could not conclude that the nebulized aqueous MgCl2 droplets crystallized to the thermodynamically stable MgCl2·6H2O [1]. What could the reason for the difference in hydrate structures of the untreated particles and those dried at 70 °C [noticable difference between the two sharp blue and red FTIR crystalline hydrate peaks (excluding the broad free water peak) at ~3500 cm⁻¹]?
During the drying of particles dispersed in air, efflorescence process is usually driven by kinetics (sometimes heterogeneous crystallization in presence of seeds), unlike the thermodynamically driven crystallization in bulk. This may cause changes in the salt-hydrate structure leading to the observation of partial deliquescence/slight water uptake at ~10-15 % RH for artificial sea-salt particles (instead of just below 33 % RH, as should be the case if MgCl2·6H2O is present). Furthermore, drying and crystallization kinetics also lead to imperfections in the crystal lattice, which may be the reason why Zelenyuk et al [2] observed rounded sub-micrometer particles of NaCl. Formation of amorphous/glass like structures as in MgSO4·XH2O and presence of mixed cation (Na+/Mg2+/Ca2+) sulfate (XH2O) in the crystalline form at high RHs (CaSO4·2H2O, ERH ≥ 90 %) have also been reported in few earlier works [3, 4]. Nevertheless, the artificial sea-salt particles obviously contain hydrates when dry and I hope that the subtle variations in structure are

covered within the estimated uncertainties of the dynamic shape factors and dry mass/density parameters and would not affect the measured hygroscopic growth factors of the particles.

References

1. Cziczo DJ, Nowak JB, Hu JH, Abbatt JPD. Infrared spectroscopy of model tropospheric aerosols as a function of relative humidity: Observation of deliquescence and crystallization. J Geophys Res — Atmos 102, 18843-18850 (1997).

2. Zelenyuk A, Cai Y, Imre D. From Agglomerates of Spheres to Irregularly Shaped Particles: Determination of Dynamic Shape Factors from Measurements of Mobility and Vacuum Aerodynamic Diameters. Aerosol Sci Technol 40, 197-217 (2006).

3. Wise ME, Semeniuk TA, Bruintjes R, Martin ST, Russell LM, Buseck PR. Hygroscopic behavior of NaCl-bearing natural aerosol particles using environmental transmission electron microscopy. J Geophys Res — Atmos 112, D10224 (2007).

4. Semeniuk TA, Wise ME, Martin ST, Russell LM, Buseck PR. Water uptake characteristics of individual atmospheric particles having coatings. Atmos Environ 41, 6225-6235 (2007).

”Revising the hygroscopicity of inorganic sea salt particles” - Response to reviewer comments -

We would like to thank again all three reviewers for their positive and constructive reviews. These changes helped to further sharpen the clarity of our manuscript. Our responses are given below (blue text).

Reviewer 1

The authors have done an admirable job of incorporating numerous comments from all three reviewers, and they have an improved and very good manuscript. The work should certainly be published, but I echo the sentiments of Reviewer 3 that although the work is "technically sound," it is "rather incremental than novel," and as such "would suggest more specialized journals." I don't think that Nature Communications is the appropriate venue for this work - it is just too specialized. I were to be asked to review this manuscript for Journal of Geophysical Research or Atmospheric Chemistry and Physics, I would unhesitatingly recommend accept immediately.

I have a few comments to propose to the authors to further strengthen this manuscript.

On p. 7 of the revised manuscript, it is stated "to convert mass growth to size growth, we proceeded as described in Zardini et al." However, this requires the reader to look up this article, and the authors could have explained what they did very simply by stating "radial hygroscopic growth was determined from mass hygroscopic growth using the assumption that the volumes of water and solute are additive," which is what Zardini assumed. That said, the authors can do better: as they know the density as a function of relative humidity (from Tang), they can actually calculate explicitly the radial growth without making the assumption that the volumes are additive. This approach would eliminate the uncertainty introduced by the assumption of volume additivity. It might not be large, but the point of the entire manuscript is that details of this nature are important. The relative importance of this assumption can be found by comparing the density of seawater with the sum (1-x)/rho_water + x/rho_seasalt (x is the solute mass fraction), which is what the density would be if volume additivity held.

Reply: We agree and thank the reviewer for this important comment. We have re-calculated the diameter growth factors from the EDB and water activity measurements as the reviewer suggested (using the dry density as measured from the helium-pycnometer and the RH-dependent density parametrisation for seawater by Tang et al.) This significantly improved the agreement between HTDMA and EDB measurements. In addition we also re-calculated the RH-dependent density assuming that the volumes of water and solutes are additive (as done before) and show the difference between the two approaches as error bars in Fig. 2c and 3. We have modified Fig. 2c, 3 and S1 and added to the method part:

”To convert mass growth to size growth we use the dry density as measured by the helium-pycnometer ($\rho_{\mathrm{dry}} = 2.017\,\mathrm{gcm}^{-3}$), while we use the RH-dependent density parametrisation of Tang *et al.*[10] for seawater. For NaCl we use $\rho_{\mathrm{dry}} = 2.16\,\mathrm{gcm}^{-3}$ and the parametrisation for the RH-dependent density of NaCl by Tang *et al.*[10]. In addition, we test the assumption that the volumes of solutes and water are additive when calculating the RH-dependent density[55] (see Table 1) and show the difference to the parametrisation of Tang *et al.*[10] as error bars (see Fig. 2c and Fig. 3).”

We modified the result part by replacing the previous sentence describing the difference between EDB and HTDMA by adding:

”The bulk values of $g_{\mathrm{e}}(\mathrm{RH})$ derived independently from HTDMA and EDB agree surprisingly well and show almost the same discrepancy compared to the values of Tang *et al.*[10] (see Fig. 2c and also Supplementary Fig. 1). The bulk water activity measurements of the inorganic sea salt are in the same range as the HTDMA and EDB measurements but exhibit a slightly smaller discrepancy compared to Tang *et al.*[10] at elevated RH (8 – 10 %).”

The authors did the extra step, which in and of itself is an important contribution to the literature, of measuring the density of sea salt, from which they obtained the value ~2.02 g/cm^3. From this value, they argued that the lower bound suggested by this reviewer in his comments on the first version of the manuscript was too low. Might the measured value provide some insight into which substance(s) contains hydrates? Is it MgCl2, or CaCl2, or Na2SO4? This would be an interesting addition to the manuscript, and I would think that some discussion of this would be value added.

Reply: From the density measurements we can not for sure define which sort of hydrates were present in the dry salt. The FTIR spectra would only give further details on the different present hydrates if reference spectra of the different possible sorts of hydrates would have been recorded, which was not the case. In addition, the deconvolution of the resulting sea salt spectra and its proper interpretation would be beyond

the scope of this work. We therefore would refrain in making any further specific claims or speculations on the sort of hydrates that were present.

A dozen lines or so before "Results," it might be advisable to mention that "hygroscopic growth" refers to diameter. This is the first mention of this in the manuscript, and although specialists in the field know that the term refers to radial growth (i.e., one dimension), a general audience won't know if this refers to mass or diameter.

Reply: We agree and have modified this sentence: "Using these three techniques, we demonstrate that the hygroscopic growth of inorganic sea salt is, in terms of diameter change, 8-15 % lower than NaCl ... "

The responses to the reviewers summarizes the work in four bullets, with two additional bullets describing what is new. This is an excellent summary of the work, which I would suggest that they incorporate into the manuscript.

Reply: We agree and have added these bullet points to the end of the discussion of the manuscript:
"To summarise:

1. The hygroscopicity of inorganic sea salt is significantly lower compared to NaCl. Therefore, hygroscopicity measurements of artificial sea salt should always be the baseline reference instead of pure NaCl.

2. Improving our understanding of the behaviour of the inorganic fraction of sea spray aerosol is a prerequisite for understanding the hygroscopicity of the complex mixture that is sea spray aerosol, including the effect of organic substances.

3. Inorganic hydrates, that are present even at low relative humidities, are clearly important for sea spray hygroscopicity.

4. Our results have relevance beyond hygroscopicity - it is common practice to estimate the organic fraction of sea spray aerosol based on their volatility (see e.g. Modini *et al.*[21]). Since most of what these authors measure may be the decomposition of hydrates this suggests this approach may be invalid (see recent work by Rasmussen *et al.*[40] on sea spray volatility)."

Finally, it is not clear in the supplemental figure 2 why the Kelvin corrections have different coefficients (-5.89 vs -4.71) in the term 1/Ddry. In the text on p. 8 I could see that the authors modified the value 6 to 4.71, but I wasn't explained where the 5.89 came from.

Reply: The 5.89 originates from the fit of the hygroscopic growth factors for NaCl calculated by us using Köhler theory (for a dilute droplet). We retrieve approximately the same values as Lewis (2006), who states in his work (see Eq. 18 in Lewis, 2006) a change in particle radius of $\triangle g(0, 0.9, \xi_\sigma) \approx \frac{-3\text{nm}}{r_{\text{dry}}}$ (note: we use diameter).
We have improved the figure caption by adding more details:
"The solid lines represent fits using the equation $g(\text{RH} = 90\%, D_{\text{dry}}) = a/D_{\text{dry}} + g_0$ (see legend for fit-coefficients). The dashed lines show the result of the Kelvin term parametrisation by Lewis[5] which uses the same fit-equation and $a = -6$ nm as a coefficient. We have used a modified version of the Lewis[5] parameterization where we use the factor $a = -4.71$ nm instead of the original value of $a = -6$ nm."

Reviewer 2

I am satisfied with the authors' reviewer responses and recommend publication.

Reviewer 3

In general, the manuscript has been thoroughly revised and the authors have responded to the reviewers' comments satisfactorily. The results provide valuable insight on a very specific but important aspect i.e. the hygroscopicity of inorganic sea-salt component in marine aerosol species. The authors claim to have established that inorganic sea-salt show 8-15% lower hygroscopic growth than pure NaCl, which has so far been considered for the parameterization of sea-salt hygroscopicity in circulation models. The work has been meticulously executed. Supporting measurements (FTIR and pycnometer) have been done to address the important question regarding dry-particle mass/density due to the possible presence of hydrates at low RH, in the revised version. In the body of the original manuscript the reference to sea-spray aerosols instead of inorganic sea-salt particles was confusing, as was rightly pointed out by Reviewer 1. It has been addressed in the revision. The authors have also done well to clarify their claims by saying that this work is a small but important step towards understanding the hygroscopic and optical properties of the more chemically complex sea-spray aerosols, which are invariably internally mixed with organics. The quality of the manuscript and clarity of purpose has improved after the revision and I recommend that the work should be published. On the other hand, I have my doubts whether it meets the novelty standards to be published in "nature-communications".

Suggested rephrasing of sentences in Pg. 5, paragraph 4: ”Inorganic sea-salt ”component contains” water even at very low RH as an integral part of the crystalline structure. ”The dominant hydrates may possibly be” MgCl2·6H2O and CaCl2·10H2O”.

Reply: We have changed this sentence as suggested:

”Inorganic sea salt components contain water even at very low RH as an integral part of the crystalline structure. Examples of hydrates which may be important are $MgCl_2 \cdot 6H_2O$ and $CaCl_2 \cdot 10H_2O$.”

Some further minor points to think about here and in reference to the modifications done in the revised manuscript i.e. regarding sea-salt particle drying in response to the questions pointed out mainly by reviewer 1 and 3 (major comments #5): How, do the authors know for sure that only the thermodynamically stable hydrates (MgCl2·6H2O and CaCl2·10H2O) are formed in dispersed particles? Are the FTIR spectra enough to conclude on these particular hydrate structures? Were the FTIR and density (pycnometer) measurements done on bulk deposited particles generated by both nebulization and from the chamber? Cziczo et al used dispersed aerosols in a flow tube for FTIR measurements and could not conclude that the nebulized aqueous MgCl2 droplets crystallized to the thermodynamically stable MgCl2·6H2O [1]. What could the reason for the difference in hydrate structures of the untreated particles and those dried at 70 °C [noticable difference between the two sharp blue and red FTIR crystalline hydrate peaks (excluding the broad free water peak) at 3500 cm-1]? During the drying of particles dispersed in air, efflorescence process is usually driven by kinetics (sometimes heterogeneous crystallization in presence of seeds), unlike the thermodynamically driven crystallization in bulk. This may cause changes in the salt-hydrate structure leading to the observation of partial deliquescence/slight water uptake at 10-15% RH for artificial sea-salt particles (instead of just below 33 % RH, as should be the case if MgCl2·6H2O is present). Furthermore, drying and crystallization kinetics also lead to imperfections in the crystal lattice, which may be the reason why Zelenyuk et al [2] observed rounded sub-micrometer particles of NaCl. Formation of amorphous/glass like structures as in MgSO4·XH2O and presence of mixed cation (Na+/Mg2+/Ca2+) sulfate (XH2O) in the crystalline form at high RHs (CaSO4·2H2O, ERH ≥ 90 %) have also been reported in few earlier works [3, 4]. Nevertheless, the artificial sea-salt particles obviously contain hydrates when dry and I hope that the subtle variations in structure are covered within the estimated uncertainties of the dynamic shape factors and dry mass/density parameters and would not affect the measured hygroscopic growth factors of the particles.

Reply: The FTIR analysis has only been performed (on the bulk salt) to check for the presence of water/hydrates in the dry salt. The analysis was not intended to reveal further physical details on the specific nature of the hydrates. We therefore decided to show the FTIR plot only in the reply comments and not in the supplement because a further more in-depth analysis would be beyond the scope of this paper. A thorough FTIR analysis would be an entire work in itself and would need further, more detailed and comprehensive FTIR measurements (e.g. recording reference spectra). This is clearly beyond the scope of this paper and possible research tasks for future work. We have clarified in the revised manuscript that the FTIR analysis was performed on the bulk salt mixture.

”We have confirmed this using Fourier transform infrared (FTIR) spectroscopy on the bulk inorganic sea salt mixture.”

We agree entirely with all the points the reviewer makes here. However, as pointed out above these are beyond the scope of our paper. As the reviewer points out, we assume that all these intricate details of hydrates are included in our measurements of the dynamic shape factor and the dry density.

References (reviewer 3):

1. Cziczo DJ, Nowak JB, Hu JH, Abbatt JPD. Infrared spectroscopy of model tropospheric aerosols as a function of relative humidity: Observation of deliquescence and crystallization. J Geophys Res - Atmos 102, 18843-18850 (1997).

2. Zelenyuk A, Cai Y, Imre D. From Agglomerates of Spheres to Irregularly Shaped Particles: Determination of Dynamic Shape Factors from Measurements of Mobility and Vacuum Aerodynamic Diameters. Aerosol Sci Technol 40, 197-217 (2006).

3. Wise ME, Semeniuk TA, Bruintjes R, Martin ST, Russell LM, Buseck PR. Hygroscopic behavior of NaCl-bearing natural aerosol particles using environmental transmission electron microscopy. J Geophys Res - Atmos 112, D10224 (2007).

4. Semeniuk TA, Wise ME, Martin ST, Russell LM, Buseck PR. Water uptake characteristics of individual atmospheric particles having coatings. Atmos Environ 41, 6225-6235 (2007).

Further changes

We harmonised the wording for GCM and use throughout the manuscript ”general circulation model” instead of ”global climate model” (both are used in the literature but the first one is more common for ECHAM). We added all editorial changes as suggested by the editor.

References

Lewis E.R., The effect of surface tension (Kelvin effect) on the equilibrium radius of a hygroscopic aqueous aerosol particle, *J. Aerosol. Sci.*, 37(11), 1605–1617, 2006.
